# Supplementary material for: Factors affecting trust in clinical trials conduct: Views of stakeholders from a qualitative study in Ghana
Source: PLOS Glob Public Health. 2023 Mar 14;3(3):e0001178. doi: 10.1371/journal.pgph.0001178 (PMC10022335; doi:10.1371/journal.pgph.0001178)
Supplement: S1 Data — (DOCX) [file pgph.0001178.s001.docx]

**In-depth Interviews (IDIs)**

**Name:** B Perceptions on the conduct of clinuical trial

<Internals\\IDIs\\A-Participated 3 or more times\\A-IDI-38yr old female-participated 3 or more times-01> - § 3 references coded [13.63% Coverage]

Reference 1 - 5.43% Coverage

Q: Do you think new drugs should be given or tried on human beings to find out how effective the drug is?

R: For me, some of the medicines have side effects and you can give it to somebody’s child and the drug can disturb the child but you can give that same drug to another person’s child and it may not disturb that child and so it depends on the person’s system because we are not the same. So if you could always check on these drugs and make sure that it is safe for the children to use them that will be fine. If the disturbances (side effects) are many, people will not be happy about that because you are helping us in terms of our health.

Reference 2 - 4.66% Coverage

Q: They usually want to try the drug and see whether the drug is good or not. So in your view, do you think such drugs they are not sure whether or not it works well, should they give it to human beings?

R: Kept quiet for some time, ok, it is good and what I can say it that it is everybody and their God (refers to luck), but well I think it is good to test them on human beings. How can I say yes or no, because if you don’t test the drug too, you will not know whether the drug is good or not and so on that side it is good to test new drugs on people (IDI-38yr old female-participated 3 or more times-01)

Reference 3 - 3.54% Coverage

Q: What is your perception about the conduct of trial to test new drugs?

R: Quite recently, there was this drug they were giving to people though I have not experienced it but they said it has killed people and disturbed other people. If it is a new drug and they don’t know how to administer it to people very well, probably somebody should have be given half of it and if thy give it to you wrongly it will disturb you.

<Internals\\IDIs\\A-Participated 3 or more times\\B-IDI-31yr old female-participated 3 or more times-02> - § 3 references coded [10.91% Coverage]

Reference 1 - 3.35% Coverage

Q: Do you think it is good to test new drugs that are yet to be given approval on human beings?

R: Yes I think they should do that because if the drug is not good for them to test on human beings they will not do that. So once the drug has come it means that it is for human being to use and I think it should be tested on human being.

Reference 2 - 2.00% Coverage

Q: Do you think the conduct of studies to test new drugs would help researchers to come out with drugs that could be used to treat diseases or not?

R: Yes it helps them to get medicines for us to use.

Reference 3 - 5.56% Coverage

Q: In this community, what do you have to say concerning the perception of people when it comes to conducting studies to test new drugs on people? Do they think it is good or not to do that?

R: For that one, some people say it is good while others say it is not good. Some of them talk about the blood they usually take from people and they think researchers take the blood and send it somewhere and use it for something and not for health reasons. They say they take the blood and use it for their own things. That is what some of the people talk about.

<Internals\\IDIs\\A-Participated 3 or more times\\C-IDI-60yr old female-participated 3 or more times-03> - § 5 references coded [12.67% Coverage]

Reference 1 - 1.44% Coverage

Q: What is the reason why they have to conduct studies to test new drugs on human beings?

R: Yes they do that in order to help us in terms of our health.

Reference 2 - 3.18% Coverage

Q: But what do you think researchers would test new drug on human beings which is not well know?

R: They use it on human being in order to improve on their health and when you also understand their work and you collect the drug, you will have good health. I have accepted to be part of that work because I knew it would help my in future.

Reference 3 - 4.42% Coverage

Q: So do you think they should test new drugs on human beings?

R: Yes, for me if they use it to work it will being about benefits to us and the benefit will be good health. They have to conduct trial studies to test the new drugs to help the people otherwise if that is not done, the world will come to an end. You come and talk to us because of diseases so that you can help us. So, if you don’t do that and you are affected with disease, you will loose your life, yes.

Reference 4 - 1.65% Coverage

Q: Do you think researchers should conduct trial studies?

R: Yes I think they should do that kind of work because if they don’t do it, it will have a negative effect on people

Reference 5 - 1.98% Coverage

Q: Does the conduct of clinical trials bring about effective drugs for the management of diseases?

R: Yes it helps you people to come out with good drugs for the management of diseases to improve on our health (IDI-60yr old female-participated 3 or more times-03)

<Internals\\IDIs\\A-Participated 3 or more times\\D-IDI-30yr old femlae-participated 3 or more times-04> - § 3 references coded [12.16% Coverage]

Reference 1 - 6.32% Coverage

Q: What is your perception about the conduct of clinical trials?

R: For such w work, I think it is important for people to be well educated to understand the rationale before they bring the work. The reason is that some people may not understand the rationale for such studies and the fact that the drug is still new, you don’t know whether if you take it will disturb you or not and that is the reason why some people don’t normally refuse to take part. For me, I think they should first of all educate people to understand the rationale of the work before the work starts (IDI-30yr old female-participated 3 or more times-04).

Reference 2 - 2.88% Coverage

Q: So do you think new drugs should be tested on human beings?

R: Left to me I would have said they should not test new drugs on human beings through the conduct of trial studies. I would have wished they test these new drugs on animals instead of human beings.

Reference 3 - 2.96% Coverage

R: Then in that sense it is good to try it on people before bringing it out for many people to use because when they just bring it into the system like that many people may lose their life as a result of using the drug and it may also not be able to cure the condition.

<Internals\\IDIs\\A-Participated 3 or more times\\E-IDI-52yr old female-participated 3 or more times-05> - § 1 reference coded [1.71% Coverage]

Reference 1 - 1.71% Coverage

R: I don’t think there is any other thing for me to talk about. For me, your work is good and it is helping us a lot.

<Internals\\IDIs\\B-Refused and dropped outs\\I-IDI-28yr old female-dropped out-04> - § 2 references coded [8.64% Coverage]

Reference 1 - 4.36% Coverage

R: Yeah, it not actually from me, it was my husband who refused and his worry was that the thing (refers to the malaria vaccine) is new and there are still piloting it and that is the reason why he has refused for the child to take the malaria vaccine and nothing more.

Reference 2 - 4.28% Coverage

Q: In your view, do you think that researchers should test new drugs on human beings?

R: Yeah, for me I will actually say yes because if it is not good they wouldn’t give it to people and I know they might have done a lot of work about it before it came out yeah.

<Internals\\IDIs\\C-Opinion leaders-Nav\\L-IDI-53yr old male Assembly Member-Nav-01> - § 1 reference coded [2.86% Coverage]

Reference 1 - 2.86% Coverage

Q: Do you think new drugs should be tested on human beings?

R: Yes, yes I believe it should be so. One thing about research work is that though there may be challenges along the line but, researchers are such that they are careful in what they do and once you are dealing with human beings, you are even extra careful and I think if such things like that (trial studies) continue, it will help us a lot because we need them, yeah. The drugs that medical officers administer to us they usually try them in so many ways and for me I don’t think there is a risk for us to do such a thing (trial studies).

<Internals\\IDIs\\C-Opinion leaders-Nav\\M-IDI-47yr old youth leader-Nav-02> - § 1 reference coded [11.85% Coverage]

Reference 1 - 11.85% Coverage

Q: So, what is the level of trust and community participation in clinical trials in Navrongo here?

R: I will say trust is high except the few people who have issues with the blood samples. But now, that thing is no more there because of trial studies conducted in the past and the benefits they have seen in those studies and the education they (refers to community members) have received from the research team and even their own community members about the usefulness of the research work.

For me, I have seen the importance of clinical trials and I will say nobody should resist taking part in trial studies. If the unfortunate happens, may be it is something that is bound to happen and not because of the trial that has caused it because we have seen a lot in Navrongo (IDI-47yr old youth leader-Nav-02).

<Internals\\IDIs\\C-Opinion leaders-Nav\\N-IDI-Paramount Chief-Nav-03> - § 1 reference coded [2.77% Coverage]

Reference 1 - 2.77% Coverage

R: Actually it is important for these trials because formerly we used to make drugs for treatment and at the same time preventives to certain diseases or illnesses without knowing. We used to just take these drugs because you are sick or you have just heard that if you take these drugs you are preventing yourself from getting attacked by this sickness or diseases. But now that these trials are going on, it is making us to know or it enlightens us to know that it has been tried and that is why the drug has been manufactured purposely because of a particular illness or disease and when you are taking it you know you are taking it because of this illness or disease. But if it fails means that may be the study has not properly been done or may be something has been omitted on the way during the trials that is why it is important to make these trials before manufacturing the drugs for the general public to use.

<Internals\\IDIs\\D-Opinion leaders-Hohoe\\O-IDI-60yr Old male Assembly Member-Hohoe-01> - § 1 reference coded [2.27% Coverage]

Reference 1 - 2.27% Coverage

R: I think they are essential element to get a perfect drug or I should say an ideal drug that can cure a number of people that is, you cannot just get start a chemical drug and begin to go mass treatment unless sample is taken, you understudy it, see the reactions if it is going to work well I think it is always important things like this are done with a sample of people for which the study and finally if they confirm, that is the essence of it.

<Internals\\IDIs\\D-Opinion leaders-Hohoe\\Q-IDI-59yr old male Assembly Member-Hohoe-03> - § 1 reference coded [3.78% Coverage]

Reference 1 - 3.78% Coverage

Q: I see, so in your view do you think it is good for researchers to test new drugs on human beings?

R: Yes! I will say yes that it is good to try because it is the human beings that are going to use it though in science they will say that those trials should have been used on animals and other yes, animals unlike the guinea pig, dogs and some other this thing, Yes so one way or the other it is the human being that are going to use the drugs so at times they go straightforward to test on the human beings but I think they go through some process before they try on human beings but what I realized is that, they don’t just try on the human being directly, stages and all those things by taking the blood samples and all those things to see how the blood effectiveness or that kind of thing before the trial on the human being yes.

<Internals\\IDIs\\D-Opinion leaders-Hohoe\\R-IDI-60yr old male opinion leader-Hohoe-04> - § 2 references coded [10.61% Coverage]

Reference 1 - 3.46% Coverage

I: What is your opinion about the conduct of clinical trials in general should new drugs be tested on human beings.

R: I think that is why it is called research, so you can administer it to about 10-20 people first to see if it would work.

Reference 2 - 7.15% Coverage

I: What is your perception about research studies that involve taking of biomedical samples (such as blood, urine, saliva etc) from participants for testing?

R: For that what I can say is those leader at the top especially the medical directors they can investigate into the diseases using blood samples to be able to tell the causes of those diseases and know what to do.

I: Please why do you think this is so?

R: I would say it is very good so we can get solutions to the various diseases.

<Internals\\IDIs\\D-Opinion leaders-Hohoe\\S-IDI-64yr old Sub-Chief-Hohoe-05> - § 1 reference coded [1.85% Coverage]

Reference 1 - 1.85% Coverage

I: Your opinion about the conduct of clinical trials.

R: I think it is dangerous because of infection (IDI-64yr old Sub-Chief-Hohoe-05)

<Internals\\IDIs\\D-Opinion leaders-Hohoe\\T-IDI-male-Forma Director of health services-Volta Region-06> - § 1 reference coded [2.23% Coverage]

Reference 1 - 2.23% Coverage

Q: First of all, may I know your views regarding whether or not there is the need for clinical trial studies to be conducted in Ghana?

R: I think it’s very necessary because looking at the situation at hand and you know the microorganisms are becoming resistance to a lot of drugs and because there are new emerging diseases, there is the need to conduct a lot more clinical trials to help address these problems.

<Internals\\IDIs\\D-Opinion leaders-Hohoe\\U-IDI-female Public Health Nurse-Volta Region-07> - § 1 reference coded [8.37% Coverage]

Reference 1 - 8.37% Coverage

Q: In your opinion do you think there is the need for clinical trials to be conducted in low income counties such as Ghana?

R: Yes because the conduct of clinical trials bring about employment and development to communities where these trials are conducted. Clinical trials are conducted in other countries and there is the need for us to also contribute to the development of new drugs and also get the benefits that come as a result of the conduct of clinical trials.

<Internals\\IDIs\\E-community members-Hohoe\\V-IDI-57yr old female community member-Hohoe-01> - § 2 references coded [8.64% Coverage]

Reference 1 - 4.79% Coverage

I: what is your opinion about the conduct of clinical trials in general, should new drugs be tested on human beings?

R: in my opinion I think these trials should be conducted because it is not all of us who are educated to understand the science behind them, so once those diseases come up, the people in front should take it up, look into it and educate us on it.

Reference 2 - 3.85% Coverage

I: So should new drugs be tested on human beings?

R: For that yes, because once it is human beings who are going to use it is necessary to try it on humans to see if it would give us some protection, then it can be administered it’s just like the yellow fever vaccine they give us it helps us.

<Internals\\IDIs\\E-community members-Hohoe\\W-IDI-29yr old male community member-Hohoe-02> - § 3 references coded [10.64% Coverage]

Reference 1 - 1.69% Coverage

I: Why are clinical trials conducted?

R: it is good because when you go to the hospital and there is the need to test your blood to see what diseases are in.

Reference 2 - 6.88% Coverage

I: what is your opinion about the conduct of clinical trials in general, should new drugs be tested on human beings?

R: I think clinical trial is bringing out a vaccine and then coming to the community to see if the vaccine would work on a particular disease. For me particularly I don’t like clinical trials and that is because as am here am very healthy then you come and tell me you have to give me some sort of vaccine, and when I come they find a particular disease with me which might rather make me to be thinking, meanwhile they are the same people giving the vaccine. So I don’t trust those things and I so I don’t want to take part.

Reference 3 - 2.07% Coverage

I: So should new drugs be tested on human beings?

R: For that yes, it is a good thing because once they have discovered a disease they need to find a cure or vaccine that could help address it.

<Internals\\IDIs\\E-community members-Hohoe\\X-IDI-44yr old female community member-Hohoe-03> - § 2 references coded [11.85% Coverage]

Reference 1 - 4.07% Coverage

I: Why are clinical trials conducted?

R: as a community we have diseases all around us so it is necessary to conduct such trials to investigate to know the particular type of diseases around us and how best to stop them.

Reference 2 - 7.79% Coverage

I: what is your opinion about the conduct of clinical trials in general, should new drugs be tested on human beings?

R: Hmmm, I have a problem with the fact that, am not sick and yet they have to give me a vaccine before I don’t really understand it, for example am not sick but I decide to paracetamol first, it doesn’t work that way and sometimes too people should be tested first before they take part in such studies.

<Internals\\IDIs\\E-community members-Hohoe\\Y-IDI-49yr old male community member-Hohoe-04> - § 1 reference coded [3.53% Coverage]

Reference 1 - 3.53% Coverage

I: what is your opinion about the conduct of clinical trials in general, should new drugs be tested on human beings?

R: I think it is necessary because it is human beings who are going to be using the vaccines, so they have to test to see if it will work or not.

<Internals\\IDIs\\F-MPs\\Z-IDI-47yr old MP-01> - § 1 reference coded [16.13% Coverage]

Reference 1 - 16.13% Coverage

Q: What is your perception on the conduct of clinical trial?

R: There are various stages that clinical trials go through. They first stage is where the drug is being tried on animals to see how it works before it is tried on human beings or people because when you first try the drug on human being and once you loss a life, you cannot bring it back and so in conducting clinical trial studies, you make sure that people don’t loss their life or get other disease as a result of their participation in the trial. Therefore, there should be a community communication strategy to educate people that it is safe for them to take part, the drug you are going to try is safe for people to use because once people are not sure of its safety, it will be difficult for them to take a decision to get involved. So communication is key when it comes to the conduct of clinical trial studies because people need to understand what you are doing and they must be convince that it will not be harmful to them when they take part and when people have that belief, they will accept it.

<Internals\\IDIs\\F-MPs\\ZZ-IDI-54yr old MP-02> - § 1 reference coded [3.63% Coverage]

Reference 1 - 3.63% Coverage

Q: What is your own perception about the conduct of clinical trial studies?

R: My own perception about the conduct of clinical trial is that it is a necessary evil and if even it is evil, it is a necessary evil. The reason is that at a certain stage, we got to carry out those trials in other to move from the sample to the population. You see, before we can carry out mass usage of the drug, we should know its effects on a smaller group and probably polish what we are having because there might be the need to improve on what we are having or to abandon it after the clinical trial. So it is we use to evaluate you know the drug and in my opinion this is very, very necessary and important yes.

<Internals\\IDIs\\F-MPs\\ZZZ-IDI-59yr old MP-03> - § 1 reference coded [8.10% Coverage]

Reference 1 - 8.10% Coverage

R: Yes, as for clinical trials, we have to do (conduct) clinical trials because we are part of the international community and we benefit from medicines that have been approved from ater a long study involving clinical trials elsewhere before they come to us here and so we are bound by the principal of reciprocity that if people are used as guinea pigs to tried such drugs and we are now benefiting from the results of such research studies we also have an obligation to also contribute to it. Now fortunately for us our educational and research institutions such as NUGOCHI and others are reputable and as a result the WHO normally wants us to participate in these things (trial studies). So the issue is that we can never over emphasize the need for us to have or conduct clinical trials because they are conducted to help settle something, and even the medicines that we take there are advantages and disadvantages then left along doing clinical trials because there will also be reasons against and reasons for it but all the same there is the need for the conduct of clinical trials.

<Internals\\IDIs\\F-MPs\\ZZZZ-IDI-49yr old MP-04> - § 1 reference coded [1.27% Coverage]

Reference 1 - 1.27% Coverage

Q: So if I got you right, you think that there is the need for researchers to conduct clinical trials?

R: Exactly, there is the need, a continues need you know *eeem* to do that you know as far as we continue to experience diseases, then there is the need to get drugs to handle them.

**Key Informant Interviews (KIIs)**

**Name:** B Perceptions on the conduct of clinuical trial

<Internals\\KIIs\\A-Clinical trials researchers\\A-KII-53yr old clinical trial investigator-01> - § 1 reference coded [3.64% Coverage]

Reference 1 - 3.64% Coverage

Q: From what you are saying it is not that clinical trials are conducted to come out with new drugs or products but even with the old ones that are in the system, clinical trials could be conducted on them to see whether they are still working well or not. Is that right?

R: Exactly and I was just talking to someone and it is a trial I am following-up on and also you know with the pneumonia vaccine, you know how we are doing it and pneumonia is still increasing and it is suggested that if the we change the schedule, the way we give it will protect children better. So we cannot get the schedule anywhere if you don’t put it into test and so though the old schedule is still going on but we have to now vary it and then look at the protection rate. So old drugs in the system are being changed to manage the same old conditions and the essence is to make work better and to be able to manage the condition well.

<Internals\\KIIs\\A-Clinical trials researchers\\B-KII-48yr old trial coordinator-02> - § 1 reference coded [2.50% Coverage]

Reference 1 - 2.50% Coverage

Q: In your opinion, do you think clinical trials or new drugs should be tested on human beings?

R: Yes it should, clinical trials should be conducted and like I said we are human beings and you just can’t start giving people things (refers to medicines) that you don’t know the effect and because we belong to different cultures and different geographical areas, I think it should be conducted within the lower and middle income countries because most of these drugs are especially the phase 1 trials are usually done in the developed world and usually on animals. So when it comes to our part of the world, it is important that we still go on with the trials to ensure that whatever we are giving to the people is safe and should not end up harming them.

<Internals\\KIIs\\A-Clinical trials researchers\\E-KII-48 yr old clinical trial Monitor-05> - § 1 reference coded [4.16% Coverage]

Reference 1 - 4.16% Coverage

**R: Okay *eeem*, it is important we conduct clinical trials because there is a burden of diseases and you know we need to prevent these diseases and for us to do that we have to use drugs and other procedures or methods to enable us confront these diseases. So clinical trials help us in finding new ways probably those methods we have been using, you have to conduct clinical trials on them to see whether they are still effective, so that is the essence because then you need to conduct clinical trial on human beings (KII-48 yr old clinical trial Monitor-05).**

Q: So what is your perception of the conduct of clinical trials or testing new drugs on human beings?

R: Yeah, what I said earlier was my personal view, if it is what is written ion the books, that is different otherwise that is my person opinion about the conduct of clinical trials.

<Internals\\KIIs\\A-Clinical trials researchers\\G-KII-55yr old clinical trial investigator-07> - § 1 reference coded [2.06% Coverage]

Reference 1 - 2.06% Coverage

Q: please, is there the need for the conduct of clinical trial studies?

**R: Yes there is the need to conduct clinical trial because we have to provide adequate data on drugs and also to ensure that it is able to treat the conditions and that is why we have to conduct clinical trials. The issue is that you cannot just give drugs without testing them (KII-55yr old clinical trial investigator-07).**

<Internals\\KIIs\\B-Ethics members\\G-KII-54yr old male ethics Ccommitteemember-Nav-01> - § 1 reference coded [4.15% Coverage]

Reference 1 - 4.15% Coverage

Q: So do you think new drugs should be tested on human being through the conduct of clinical trials to find out their efficacy level?

R: When you say clinical trials, the research goes through some stages and those studies should give us information as to why you have to go further to conduct the clinical trial. If you have done the initial studies and realized that the drug can never be used on human beings, why would a researcher advise that the drug should be used on human beings, no because there is a narrow range of acceptability that can lead to a certain combination for the drug to be considered as far as clinical trial is concerned.

<Internals\\KIIs\\B-Ethics members\\H-KII-47yr old male ethics committee member-Nav-02> - § 1 reference coded [2.42% Coverage]

Reference 1 - 2.42% Coverage

Q: so you don’t have any problem when it comes to the conduct of clinical trials and your perception is that it is positive and it should be encouraged?

R: **yes, to me it should be encouraged, if only it is done you know there are rules governing every procedure and I know clinical trial also has its own rules that people have to study and go by that rule so if it is followed very well to the extent that certain errrhhh human subjects will not be affected in any way or even if they will be affect in extend there is a way of alleviating their suffering or compensating for them as they agree to be part of it then there shouldn’t be a problem (KII-47yr old male ethics committee member-Nav-02)**

<Internals\\KIIs\\B-Ethics members\\I-KII-42yr old female ethics committee member-GHS-03> - § 2 references coded [3.06% Coverage]

Reference 1 - 1.70% Coverage

Q: As an ethics review committee member, what is your perception about the conduct of clinical trial studies?

R: When you say the conduct of clinical trial what exactly do you mean because we as ethics members are supposed to review and approve research protocols and so what exactly do you mean when you say conduct?

Reference 2 - 1.37% Coverage

Q: Do you thing trial studies should be conducted in Ghana?

R: Yea it should be encouraged because it is helpful, without trial studies we are not also going to get medicine and the things we need in the system but it however has to be done the right way (.

<Internals\\KIIs\\B-Ethics members\\K-KII-51yr old female ethics committee member-Nav-05> - § 1 reference coded [2.22% Coverage]

Reference 1 - 2.22% Coverage

Q: Do you think researchers should conduct clinical trial studies to test new drugs?

R: yea h but if you don’t conduct it, who should be guinea pig for another person to benefit? The issue is because populations are different, you have to conduct clinical trial to know whether the new drug works in your population. There are so many dynamics to the working of the drugs and that is why drugs are introduced usually in another continent for example, they have to try it in a few places first.

<Internals\\KIIs\\C-FDA members\\L-KII-35yr old female-FDA-01> - § 1 reference coded [4.25% Coverage]

Reference 1 - 4.25% Coverage

Q: Is there the need for clinical trials to be conducted in low-income countries such as Ghana?

R: Yes there is the need because it is through clinical trials we are able to improve drug therapies, existing procedures or bring about new innovative procedures to address certain diseases that are sometimes specific in our country. For instance if it is a cycle cell drug that is being developed it will help because circle cell is mainly found in African and Asia and in Europe, it is not a problem and so it is no much of a concern to them. So, clinical trials that will help to improve on our health if we conduct them it would be better than always depending on information from outside. If we conduct good clinical trials here it would help us gather quality data to inform policy decision and also help us to make better judgment in terms of health care management.
