# Supplementary material for: Factors affecting trust in clinical trials conduct: Views of stakeholders from a qualitative study in Ghana
Source: PLOS Glob Public Health. 2023 Mar 14;3(3):e0001178. doi: 10.1371/journal.pgph.0001178 (PMC10022335; doi:10.1371/journal.pgph.0001178)
Supplement: S2 Data — (DOCX) [file pgph.0001178.s002.docx]

**In-depth Interviews (IDIs)**

**Name:** inadequate informed consenting procedures

<Internals\\IDIs\\A-Participated 3 or more times\\A-IDI-38yr old female-participated 3 or more times-01> - § 2 references coded [10.57% Coverage]

Reference 1 - 6.50% Coverage

Q: Concerning the research work and the clinical trials they recruit people into at the community level, would you say the way they explain the work to people before they will take a decision to be part of it is also a problem that makes people to refuse to take part in the work because the researchers are not able to explain the things very well for people to understand?

R: Yes, that is also a factor and it is possible because it has happened to me before and sometimes the way they ask their questions is not right, they ask whether you drink, smoke and whether you have eaten or not, ok fine that one I will tell them I have not eaten. Asking whether you smoke or not do you want to know people who smoke, so that you will go and be making fun of them in your offices.

Reference 2 - 4.07% Coverage

Q: I am taking about how the research team administers informed consent before they recruit people into clinical trial studies. Does that also affect trust and decision of people to take part in trial studies?

R: For that one I cannot say much because they are different people who do that and for those who recruited my child I did not see any problem with that because I understood everything they had explained and where it is not clear to me, I asked them for further explanation.

<Internals\\IDIs\\A-Participated 3 or more times\\D-IDI-30yr old femlae-participated 3 or more times-04> - § 1 reference coded [3.45% Coverage]

Reference 1 - 3.45% Coverage

Q: What of informed consent administration, do you think where the field staff is not able to explain the rationale of the work very well to the person it may affect the person’s participation?

R: Yes it is also a problem especially the older people, if they don’t understand the issues they will not participate.

<Internals\\IDIs\\B-Refused and dropped outs\\F-IDI-28yr old female-Dropped out-01> - § 3 references coded [14.95% Coverage]

Reference 1 - 6.85% Coverage

Q: You said when you go to the hospital they will collect the child’s folder and be taking pictures with it and they will not tell you the reason why…..

R: Yes you know when you finish everything and now you are about to go to dispensary and collect drugs, they will collect the folder and whether they will take the picture or they will write what and they will not tell you why they are doing that and when they finish they now give the folder back to you to go for your medicine. Anytime you go they keep doing the same thing, they will take a picture of what the doctor wrote during the consultation for that day and I don’t know why I should keep bringing my child’s folders anytime the child is sick for you to take a picture of what the doctor wrote and you are not telling me what you are using it for.

Reference 2 - 2.85% Coverage

Q: Before they recruited your child to be part of the study did they tell you that they would do all those things you mentioned?

R: It is in the form and I actually asked the one who recruited my child whether they will do all those things and he said yes and that made me to join and they are not doing or going by what is in the form.

Reference 3 - 5.25% Coverage

R: I did not know the reason he was giving and that was why I refused. He said they would take the blood and test to know the sickness the child has and for them to treat it and I was like if the child is sick and you are taking the blood to go and check, I don’t have any problem with that but where the child is not sick and yet you want to take her blood, that one I will not agree.

Q: But you said the person was not able to explain the reason why they take the blood sample?

R: The one who recruited us told me that she is just to administer the consent form and recruit me because she does not know much about it.

<Internals\\IDIs\\C-Opinion leaders-Nav\\L-IDI-53yr old male Assembly Member-Nav-01> - § 1 reference coded [3.68% Coverage]

Reference 1 - 3.68% Coverage

Q: Do you think informed consent is also a factors that can affect trust and participation in trial studies of it is not properly done?

R: Yes it is a factor if not properly done can bring about mistrust. Some of the people are such that when you come and you don’t translate the consent form properly to them and he/she aggress and signs or thumbprint, then later on he comes to realize that some of the things you are doing are going into he/her privacy, you see them trying to back out. So the consent form is one other thing that will bring about trust or distrust depending on how it is done by your workers. So it should properly be translated by somebody who understands the English and the local language for them to understand it better and to be able to accept it.

<Internals\\IDIs\\D-Opinion leaders-Hohoe\\T-IDI-male-Forma Director of health services-Volta Region-06> - § 1 reference coded [2.99% Coverage]

Reference 1 - 2.99% Coverage

Q: What of informed consent, does it affect trust and participation in the conduct of trial studies?

R: It may be because in an attempt to get many people enrolled into the trial, they (study workers) may not even explain the issues to the subjects for them to really understand and actually make an informed decision to be part of the study. So it is possible because in an attempt to recruit people into the study the fieldworkers may give different information to the participants, which may not be in the consent form, so it is possible it may affect.

<Internals\\IDIs\\F-MPs\\ZZ-IDI-54yr old MP-02> - § 1 reference coded [2.55% Coverage]

Reference 1 - 2.55% Coverage

R: The informed consent is very necessary and once it is done properly it should influence decision of people positively because this is a trial and this is a person who is not sick, there is no emergency about it and so the person’s concern and understanding the process is very necessary to ensure a positive outcome. Once the person feels that there is something he/she does not understand, the person is likely along the line to break the rules of the trial and that is not what we want.

<Internals\\IDIs\\F-MPs\\ZZZZ-IDI-49yr old MP-04> - § 1 reference coded [3.95% Coverage]

Reference 1 - 3.95% Coverage

**Use text**

Q: What of informed consent? Do you think it plays a role to the issue of trust and distrust?

R; Yes it does and earlier I said that depending on how these things are explained to the people, *aaah* it affects the level of trust. If really you explain to me very well that this is what you are doing and this is the nature of it….once I understand and through that process of explaining things to me, you reduce my fears, then you are building my trust level for that trial and whatever drug you are going to use (test) as compare to where I am just there and then you come and say you want to take my blood or want me to take part in this trial, I will be afraid. So explaining things to me in the form of consent and ensuring that I agree and when I understand you I say oh I agree, I consent to whatever you are doing. So really, informed consent increases trust level.

**Name:** lack of education & engagment & no understanding

<Internals\\IDIs\\A-Participated 3 or more times\\C-IDI-60yr old female-participated 3 or more times-03> - § 1 reference coded [5.75% Coverage]

Reference 1 - 5.75% Coverage

Q: Would you say that clinical trial researchers have not been able to adequately explain or educate people on the reasons for the conduct of clinical trials and that is why some people will not want to take part?

R: Yes it is possible, it is possible because the one who came to me this morning before you came; you have been able to explain the things for me to understand. Some of your workers are there, they are not able to explain the things like you have done and that is the way it is and once the person have not understand the issues, he/she will say oh this VAST people can disturb *ooo,* laughter, yes.

<Internals\\IDIs\\A-Participated 3 or more times\\D-IDI-30yr old femlae-participated 3 or more times-04> - § 1 reference coded [1.97% Coverage]

Reference 1 - 1.97% Coverage

Q: Do you think lack of education make people not to take part in such studies in this community?

R: Yes because if you do not understand the work, you may not want to take part (IDI-30yr old female-participated 3 or more times-04).

<Internals\\IDIs\\B-Refused and dropped outs\\H-IDI-32yr old Female-Refused to take part-03> - § 1 reference coded [5.10% Coverage]

Reference 1 - 5.10% Coverage

So trial drugs like that the education is not always done well and when people take them and get such problem, it scars other people away from similar activities. Interventions like that people are usually taking the drug the way they want, which is partly because of lack of education on how people could use the drugs. Many people died as a result of taking that drug especially in the East.

<Internals\\IDIs\\B-Refused and dropped outs\\I-IDI-28yr old female-dropped out-04> - § 1 reference coded [7.25% Coverage]

Reference 1 - 7.25% Coverage

R: The issue is that the people are afraid already and so there was supposed to be public education on it (refers to the malaria vaccine), a detailed one because some of the health workers don’t explain it very well to the people, they will only tell them that *oooh* it is a new malaria drug and then they want to give it to your child. They don’t give them information such as how it came about, the effects, how it will work and all those things (IDI-28yr old female-dropped out-04)

<Internals\\IDIs\\C-Opinion leaders-Nav\\L-IDI-53yr old male Assembly Member-Nav-01> - § 1 reference coded [2.72% Coverage]

Reference 1 - 2.72% Coverage

Q: Do you think lack of proper education or sensitization can also affect trust of people when it comes to the conduct of clinical trials?

R: Yes sensitization is one because when the people are not well educated, they will not have the trust because what they know is that when you take that drug, you become impotent and so you need information to be able to overcome that particular perception.

Those who also have their religious beliefs contrary to these things, when they are educated properly, they will know that religion has nothing to do with research studies.

<Internals\\IDIs\\C-Opinion leaders-Nav\\N-IDI-Paramount Chief-Nav-03> - § 1 reference coded [0.79% Coverage]

Reference 1 - 0.79% Coverage

R: But for a smooth study or a better study you have to follow up as the concerned investigator. Or as a concerned investigator you have to follow up to make sure your study is approved. But if you feel a bit lazy in your study then you cannot end up very well.

<Internals\\IDIs\\D-Opinion leaders-Hohoe\\S-IDI-64yr old Sub-Chief-Hohoe-05> - § 1 reference coded [4.52% Coverage]

Reference 1 - 4.52% Coverage

I: What are the factors influencing trust and the decision to participate in trial studies?

R: First of all is education, if you educate me well to know the benefits of it, then when I hear from a friend that it is good then I will also get involved (IDI-64yr old Sub-Chief-Hohoe-05)

<Internals\\IDIs\\D-Opinion leaders-Hohoe\\T-IDI-male-Forma Director of health services-Volta Region-06> - § 1 reference coded [2.15% Coverage]

Reference 1 - 2.15% Coverage

Q: Would you say is because it was ebola otherwise if it were any other trial, it would have been conducted?

R: Yea the name ebola was also part of the problem and I am always of the view that if the people really understand clinical trials, if they really know what it’s involves, ebola or no ebola, they will still take part yea. The ebola trials have been conducted in other African Countries (IDI-male-Forma Director of health services-Volta Region-06).

<Internals\\IDIs\\D-Opinion leaders-Hohoe\\U-IDI-female Public Health Nurse-Volta Region-07> - § 1 reference coded [8.89% Coverage]

Reference 1 - 8.89% Coverage

Q: What other factors do you think can affect trust and participation of people in clinical trials?

R: If the people don’t understand what exactly they want to do, if the information does not go down well with them they will not agree to take part. You know you have to weigh the positives and negative and it is through the information they give that you will be able to do that and then take a decision whether or not to take part. So if that is not done then it can affect participation (IDI-female Public Health Nurse-Volta Region-07).

<Internals\\IDIs\\E-community members-Hohoe\\X-IDI-44yr old female community member-Hohoe-03> - § 1 reference coded [3.90% Coverage]

Reference 1 - 3.90% Coverage

I: What are the factors that affect trust and decision to participate in trial studies?

R: I think once education is done well and the people understand what is going on then that could motivate them to partake.

<Internals\\IDIs\\E-community members-Hohoe\\Y-IDI-49yr old male community member-Hohoe-04> - § 1 reference coded [5.12% Coverage]

Reference 1 - 5.12% Coverage

I: How does trust affect the conduct of clinical trials?

R: for trust it does a long way to affect the conduct of clinical trial because I don’t think they involved the chiefs and elders with what they wanted to come and do and even the community members were not really involved so there was no trust in the trial. Education plays an important role in conducting such studies (IDI-49yr old male community member-Hohoe-04).

<Internals\\IDIs\\F-MPs\\Z-IDI-47yr old MP-01> - § 2 references coded [24.33% Coverage]

Reference 1 - 16.13% Coverage

Q: What is your perception on the conduct of clinical trial?

R: There are various stages that clinical trials go through. They first stage is where the drug is being tried on animals to see how it works before it is tried on human beings or people because when you first try the drug on human being and once you loss a life, you cannot bring it back and so in conducting clinical trial studies, you make sure that people don’t loss their life or get other disease as a result of their participation in the trial. Therefore, there should be a community communication strategy to educate people that it is safe for them to take part, the drug you are going to try is safe for people to use because once people are not sure of its safety, it will be difficult for them to take a decision to get involved. So communication is key when it comes to the conduct of clinical trial studies because people need to understand what you are doing and they must be convince that it will not be harmful to them when they take part and when people have that belief, they will accept it.

Reference 2 - 8.20% Coverage

R: That is why I earlier on indicated that researchers must show that it is safe for people to get involve in whatever trial they want to undertake by showing pictures and assure people that it has been done in other places and there are the negative or positive effects. The issue is that it is a very high risk for you to take part in trial studies especially, phase one clinical trials.

….So for me, I will not even take a decision to take part in the initial stages of the trial (refers to phase one trials) because of the high risks involve (IDI-47yr old MP-01).

<Internals\\IDIs\\F-MPs\\ZZ-IDI-54yr old MP-02> - § 1 reference coded [5.56% Coverage]

Reference 1 - 5.56% Coverage

Q: What of community engagement and sensitization when it comes to the conduct of clinical trials. Do you think it can also affect the conduct of trial studies?

R: Yes the transparency of the process, general education of the public, involvement of focal persons and key individuals for example if a doctor is ready to subject himself to it, a professor is ready to subject him/herself to it can affect other people decision positively. However, if all these type of people raise question marks and are not ready to subject themselves to the trial then along the line people will start raising questions why all these individuals are not supporting this trial and now you want me to participate.

So I think that the way you educate people during community entry process, the involvement of assembly members, the gatekeepers you know is all part of the process. You got to ensure that you involve all of them including the district assemblies, the health directorates; they should all get involved to understand the process very well before you finally hit the grounds.

<Internals\\IDIs\\F-MPs\\ZZZZZ-IDI-49 yr old MP-05> - § 2 references coded [8.78% Coverage]

Reference 1 - 5.55% Coverage

R: It is very low because of the credibility gap, in the past people have abused people they have come into community, not entering the community properly, not educating the community people properly, not telling them exactly what the consequences of the things are and sometimes it is distasteful so it went quite, sometimes back fired and it has created problems. So today when you come and you are talking they want to be sure is it all the information I need to know or you are hiding something so the suspicion is very high and I think the only way to eradicate this suspicion is to be consistent and truthful. And we as a country and for that matter ministry of health must have a protocol that is known to the people so that I know that once it is going to be a trial in my constituency I expect A, B, C, D, E, F is going to happen. Once I know that it will happen in Asewase, it will happen in Bolga, it will happen in Zebila, the same thing is happening in Ho, the same thing is happening in Kpando, or Sefwi or somewhere. Then this suspicion will be off but if you enter the community differently and behave differently and give half information, in some cases you give full information, the protocol is not well known definitely the suspicion will remain.

Reference 2 - 3.23% Coverage

R: Obviously, if you don’t introduce yourself properly, if you don’t enter the community properly, you know in every community there are the champions, they are spoilers you don’t try to get the buy in from them, let them appreciate and understand so that they can explain to the illiterates in those communities, oh this is about it. They will tend to trust better than you the one coming so entering the community like I told you earlier is a very important thing because it helps avoid this thing and like you said in a community where they have done one or two trials, they are familiar with it as compared to those who have never done it. So entering the two communities will be different, the approach will be completely different.

**<Internals\\IDIs\\F-MPs\\ZZ-IDI-54yr old MP-02> - § 1 reference coded [4.30% Coverage]**

Reference 1 - 4.30% Coverage

R: Also if you want to conduct clinical trial in Ghana the clients or people would want to know whether that particular trial has been conducted elsewhere or not and what has been the results or whether they are the first people that are going to be subjected to such trial!. Secondly what is the rationale of such a trial and how will it benefit them as participants and the whole society and of cause the safety because they are really interested in the safety because em some of them remember that when they were young they were subjected to certain vaccinations and probably they reacted violently (refers to negatively), they reacted so many ways after they were vaccinated and it is this fear that they consider in trials that are coming in and they ask themselves am I going to get such reactions again and so on, yeah (IDI-54yr old MP-02).

**Name:** risk-fear of possible harm from new drugs

<Internals\\IDIs\\A-Participated 3 or more times\\A-IDI-38yr old female-participated 3 or more times-01> - § 1 reference coded [9.54% Coverage]

Reference 1 - 9.54% Coverage

Secondly, there was a time the nurses brought malaria drug and said it was a new medicine for me to collect and give to the child and I told them no because I don’t know how the drug will work. They told me the drug would not disturb the child and even if it disturbs the child I should bring the child to the hospital and explain that they (nurses) gave the child medicine and as a result, the child got these side effects and if I say that they will treat the child. I agreed and gave the medicine to the child and for God and man the child was vomiting and running diarrhea and at a point the child became very weak. I had to take the child to the hospital and they gave the child drips and the vomiting and the diarrhea all stopped. It was the same thing that happened with my step daughter’s child, he also run diarrhea and became very weak and she also took the child to the hospital before all those things (refers to the vomiting and the diarrhea) stopped. So for us, we have not been collecting that medicine again and the same for other people in this community whose children took the drug and experienced the same side effects.

<Internals\\IDIs\\A-Participated 3 or more times\\C-IDI-60yr old female-participated 3 or more times-03> - § 1 reference coded [2.60% Coverage]

Reference 1 - 2.60% Coverage

Q: Do you think because of the side effects of using new drug make people not interested in taking part in such studies in this community?

R: Yes some people can refuse because of side effects but I cannot be able to say exactly what the issue is once I am not in their mind.

<Internals\\IDIs\\B-Refused and dropped outs\\G-IDI-23yr old female-Refused to take part-02> - § 1 reference coded [8.98% Coverage]

Reference 1 - 8.98% Coverage

R: Yea it is a reason because I have even experienced it with this my boy. When I gave the drug to him, his body became so hot and he could not even go to toilet for the whole of that week. When I took him to the hospital, they did lab test and nothing was wrong with him and it now occurred to me that is like because of the drug that his body is hot.

<Internals\\IDIs\\B-Refused and dropped outs\\H-IDI-32yr old Female-Refused to take part-03> - § 2 references coded [21.87% Coverage]

Reference 1 - 15.62% Coverage

Q: Yes and I wanted to find out why the problem is that made you to refuse.

R: It was my husband that refused because the time I gave birth he came around for the child’s naming ceremony and after that he strayed for some time before he went back. According to him, he had information from audio recording that they usually bring these medicines to test it on us and see whether they are good or not. Somebody actually sent the audio recording to him and now that he has heard something concerning the malaria vaccine, then he rejected it and said the child would not take it. I personally talked to him to allow the child to receive the vaccine and he did not try to understand me and because I did not also want to force him, I had to stop. In the video, the voice was a woman’s voice and what she said was that they tested the injection on dogs and the dogs died, they tested it on some human beings and they also died and so people should be careful with medicines that they brings into the country and ask people to take. She said these are medicines they usually want to try and see so, if they test and many people die from using it, then they will not use it again. That was the content of the audio.

Reference 2 - 6.25% Coverage

R: Yes for that one, it is a factor because people talk about it especially this malaria drug. When it came people were not ready for it and I have heard that from the mouth of many people. They are saying that the research people are sitting there and collecting vaccines from wherever and come and inject and kill children. The reason why some people have that perception is that you see this elephantiasis drug, when it first came, many people who took it died and it was not easy (IDI-32yr old Female-Refused to take part-03).

<Internals\\IDIs\\D-Opinion leaders-Hohoe\\O-IDI-60yr Old male Assembly Member-Hohoe-01> - § 2 references coded [4.82% Coverage]

Reference 1 - 1.52% Coverage

So well it is the fear, it is fear, the anxiety because of the story we hear that is why probably when you are doing you will have problems. But looking at things, the educated ones, I know there is need for a thing like this is a matter of explanation to them.

Reference 2 - 3.30% Coverage

Q: ok so they didn’t have any issue but the trial didn’t come on, I mean subsequently the trial was suspended*.* In your opinion what will you say was the key reason why it was suspended?

R: well like I said earlier on, you know the risk factor is what when people begin to hear things that sometimes when you want to do it many people are not giving themselves up and so if they are not giving themselves up, it become difficult for the trial to be conducted When you need ten people out of about hundred and then only one is coming, it is not effective here and it will not give you the result probably that is the reason why it could not been conducted.

<Internals\\IDIs\\D-Opinion leaders-Hohoe\\P-IDI-65yr old female Assembly Member-Hohoe-02> - § 1 reference coded [1.23% Coverage]

Reference 1 - 1.23% Coverage

R: You may faint or you may have skin rashes and those things yah.

Q: Okay so as a result of the drug?

R: Yes, because you didn’t take our blood to the Lab and see that this medicine is stronger than this person’s blood.

<Internals\\IDIs\\D-Opinion leaders-Hohoe\\Q-IDI-59yr old male Assembly Member-Hohoe-03> - § 2 references coded [8.59% Coverage]

Reference 1 - 5.48% Coverage

R: yes, you see the only problem I foresee is that you see the outbreak before you know they were trying to do the trial you know the outbreak scared a lot of people because when you watched the television and then you hear on radio the education that is ongoing, it scared a lot of people, that is why they were not able to, you know convince the people. The way *ebola* one came up and people were dying and maybe they said if you touch somebody that kind of thing even I myself was scared. If there weren’t an outbreak and they were not showing the way people were dying and those things ah the could have succeeded in conducting the trial (IDI-59yr old male Assembly Member-Hohoe-03)

and when they started the education and all those things people started scaring a lot of people and that if you are not very careful and they try it and then there is an outbreak then the whole community will suffer if not because of the outbreak unlike we all know that malaria the outbreak is there but we are not scared about malaria but

the way *ebola* one came up and people were dying and maybe they said if you touch somebody that kind of thing even I myself was scared, it scared a lot of people about the whole issue and now that they want to try the thing on you, you know nobody yes so if there were not to be an outbreak but they know that there is Ebola oh but not on even a large scale whereby the whole world got to know about Ebola they were showing it, the way people were dying and those things ah the could have succeeded in making the trial.

Reference 2 - 3.11% Coverage

Q: I see but it is just that the Ebola thing was a different case

R: yes it was different issue all together because it scared a lot of people and that the even we didn’t understand the whole thing even the trial we didn’t understand it and the way they saw it all of a sudden people are dying we cannot shake hands with people and all those things and they say they are coming to do trial, we thought they are bringing some sample to even they didn’t start any trial and there was a hullabaloo that oh they have already tried it on some people and that kind of thing so everybody was scared that was the reason why it could not succeed otherwise it could have been easily conducted.

<Internals\\IDIs\\D-Opinion leaders-Hohoe\\T-IDI-male-Forma Director of health services-Volta Region-06> - § 1 reference coded [5.00% Coverage]

Reference 1 - 5.00% Coverage

R: The main concern of people is safety and how trials would benefit them. These are the issues I have heard so far *ehaaa*. For instance when we wanted to introduce this new malaria vaccine, you know those issues came up again and they called me on radio station and asked me that why is it that when we are doing those things, they use us as guinea pigs and things like that. So they called me from the radio station to explain what the whole thing is about and I had to explain and then assure the public that the thing is safe. So most at times safety is actually the main complain because those who are against the thing (trials) preach that to the people, that is what they preach that it is not safe, you know it would damage the people and that is why they would not do it in Europe or America and they want to use our people as guinea pigs. As far time, people are not really care about that, the major thing is the safety (IDI-male-Forma Director of health services-Volta Region-06).

<Internals\\IDIs\\D-Opinion leaders-Hohoe\\U-IDI-female Public Health Nurse-Volta Region-07> - § 1 reference coded [6.59% Coverage]

Reference 1 - 6.59% Coverage

Q: Do you think time and side effects of new drugs also affect participation in clinical trials?

R: Yes it can affect the conduct of trial studies because if you don’t communicate it well the people will not understand. You know community participation, if you involve the community they will give you the time for you to come and meet with them on what you want to do.

<Internals\\IDIs\\E-community members-Hohoe\\V-IDI-57yr old female community member-Hohoe-01> - § 1 reference coded [7.63% Coverage]

Reference 1 - 7.63% Coverage

I: What are the factors that generally affect the conduct of trial studies in this community/Ghana?

R: when these studies come, sometimes you want to see someone who has taken part and then once the person comes and you see the result you also take part. So from there you can tell the risks and benefits of the trial then once I know all that then I can join myself (IDI-57yr old female community member-Hohoe-01).

And am sure that is one of the reasons the community will look at to join such trails. Also I think community engagement should be enforced because once the health authorities know they should come and involve us.

<Internals\\IDIs\\E-community members-Hohoe\\W-IDI-29yr old male community member-Hohoe-02> - § 1 reference coded [3.94% Coverage]

Reference 1 - 3.94% Coverage

I: What were the perceptions of community members about the ebola trial?

R: for the community the people believed that it was not a disease that would come so there is no way they would take part in such a trial. And that is because some felt that was if they take part and realize that someone in the community had the virus then it could have caused fear and panic.

<Internals\\IDIs\\F-MPs\\ZZ-IDI-54yr old MP-02> - § 1 reference coded [4.30% Coverage]

Reference 1 - 4.30% Coverage

R: Also if you want to conduct clinical trial in Ghana the clients or people would want to know whether that particular trial has been conducted elsewhere or not and what has been the results or whether they are the first people that are going to be subjected to such trial!. Secondly what is the rationale of such a trial and how will it benefit them as participants and the whole society and of cause the safety because they are really interested in the safety because em some of them remember that when they were young they were subjected to certain vaccinations and so on and they reacted violently (refers to negatively), they reacted so many ways after they were vaccinated and it is this fear that they consider in trials that are coming in and they ask themselves am I going to get such reactions again and so on, yeah.

<Internals\\IDIs\\F-MPs\\ZZZ-IDI-59yr old MP-03> - § 1 reference coded [3.84% Coverage]

Reference 1 - 3.84% Coverage

Q: Do you also think that the risks involved in clinical trials to influence that decision to take part?

R: Yes, what I am saying is that you have to be as frank as possible with people and with medicines, there are side effects and so for it to advance from animals to human beings then you must demonstrate to the person that when the drug was tried in animal and other human beings, these were the side effects but then they were minimal. So that must be established and if there is evidence of it to show, fine.

<Internals\\IDIs\\F-MPs\\ZZZZ-IDI-49yr old MP-04> - § 1 reference coded [3.66% Coverage]

Reference 1 - 3.66% Coverage

Q: What of uncertainty, side effects and capacity of trial team, do you think these also influence trust of people negatively of positively?

R: You mean the drugs will work or is good for them or?

Q: Yes and may be the side effects. Progressing

R: The fear and the risks involved you know, yes these are factors that can influence trust especially the side effects, once they are not very certain of what they are taking, *aaah*, the fear is there and because the fear is there, the trust is low because the person is so afraid that ooo this thing will rather lead him/her to other conditions or harm him/her and that is where the issue of trust begins and if I’m not sure of what this drug would do then I don’t trust it. So I agree that uncertainty about the kind of drug they are taking would definitely affect trust and participation, yes (-IDI-49yr old MP-04).

<Internals\\IDIs\\F-MPs\\ZZZZZ-IDI-49 yr old MP-05> - § 3 references coded [6.16% Coverage]

Reference 1 - 0.56% Coverage

R: The bottom line in trials are that sometimes the people that are going to go through that you think when you do you will die.

Reference 2 - 2.98% Coverage

R: The participant sometimes just think if it goes on you are going to die so that is why I am saying if there is a proper protocol that says that you explain to the people, the team leader must have say some minimum qualification and those who are going to administer it must have such, and everybody knows. So when you come, who is your team leader can I see your qualification and people are looking at it based on the standard that has been set then they are able to satisfy themselves easily. But when it is just that oh, we are here it will help us, you are only mentioning the benefits without mentioning the possible consequences obviously you are going to create problems.

Reference 3 - 2.61% Coverage

R: You see when ebola comes and even by just starting an ebola person this is the consequences you need to educate them properly. Educating the people properly about why you want to do it, what are the benefits the consequences would take more time and energy than if there was not an outbreak because everybody saw the consequences of the outbreak in Sierra Leone and Liberia. And obviously you just mention the name and it is now to scare people to their spine so I think that, that was partly the reason because of the escalation of the news about the terrible effect of the ebola and all that.

**<Internals\\IDIs\\F-MPs\\ZZ-IDI-54yr old MP-02> - § 1 reference coded [4.30% Coverage]**

Reference 1 - 4.30% Coverage

R: Also if you want to conduct clinical trial in Ghana the clients or people would want to know whether that particular trial has been conducted elsewhere or not and what has been the results or whether they are the first people that are going to be subjected to such trial!. Secondly what is the rationale of such a trial and how will it benefit them as participants and the whole society and of cause the safety because they are really interested in the safety because em some of them remember that when they were young they were subjected to certain vaccinations and so on and they reacted violently (refers to negatively), they reacted so many ways after they were vaccinated and it is this fear that they consider in trials that are coming in and they ask themselves am I going to get such reactions again and so on, yeah.

**Name:** Rumour and the use of internet can influence trust

<Internals\\IDIs\\B-Refused and dropped outs\\H-IDI-32yr old Female-Refused to take part-03> - § 1 reference coded [15.62% Coverage]

Reference 1 - 15.62% Coverage

Q: Yes and I wanted to find out why the problem is that made you to refuse.

R: It was my husband that refused because the time I gave birth he came around for the child’s naming ceremony and after that he strayed for some time before he went back. According to him, he had information from audio recording that they usually bring these medicines to test it on us and see whether they are good or not. Somebody actually sent the audio recording to him and now that he has heard something concerning the malaria vaccine, then he rejected it and said the child would not take it. I personally talked to him to allow the child to receive the vaccine and he did not try to understand me and because I did not also want to force him, I had to stop. In the video, the voice was a woman’s voice and what she said was that they tested the injection on dogs and the dogs died, they tested it on some human beings and they also died and so people should be careful with medicines that they brings into the country and ask people to take. She said these are medicines they usually want to try and see so, if they test and many people die from using it, then they will not use it again. That was the content of the audio (IDI-32yr old Female-Refused to take part-03).

<Internals\\IDIs\\C-Opinion leaders-Nav\\L-IDI-53yr old male Assembly Member-Nav-01> - § 2 references coded [4.55% Coverage]

Reference 1 - 3.05% Coverage

Q: What are the factors that affect the conduct of clinical trials in low income countries like Ghana?

R: You know the recent events happening in our part of the world is likely to affect that kind of thing (the conduct of clinical trials) especially the recent situation where people are going about saying that they are bringing drugs for children to take and die and all of that, you know there is that fault information in the system. So that is the only risk and we need to intensify our campaign otherwise people can also come with their agenda that can negatively affect the prospectus of your research centre and your work (IDI-53yr old male Assembly Member-Nav-01)

Reference 2 - 1.49% Coverage

**Solution**

So I want to say that in other to overcome such a challenge, you need to really maintain and let people know where and how you started so that if fault people are also entry into the community, people will be able to make that distinction and know that they are not the people they have been working with.

<Internals\\IDIs\\C-Opinion leaders-Nav\\M-IDI-47yr old youth leader-Nav-02> - § 1 reference coded [12.32% Coverage]

Reference 1 - 12.32% Coverage

Q: Apart from the blood issue that you talked about, are there some community members who still do not trust our research activities especially the trial studies and what probably is the reason why they don’t trust?

R: I would say some of the educated people are rather trying to pollute the minds of some community members because they (the educated people) have traveled wide and they think what is done in other areas should not be applied here. I don’t think you can go to Europe and just conduct clinical trial like that it would involve a lot of money. So the educated people will say that no, no don’t volunteer you blood or anything like that to researchers, do you know that there are risks? So, whatever the person (educated person) will tell the family member, it would get out there and pollute the minds of other people (IDI-47yr old youth leader-Nav-02).

<Internals\\IDIs\\D-Opinion leaders-Hohoe\\Q-IDI-59yr old male Assembly Member-Hohoe-03> - § 2 references coded [8.88% Coverage]

Reference 1 - 5.48% Coverage

R: yes, you see the only problem I foresee is that you see the outbreak before you know they were trying to do the trial you know the outbreak scared a lot of people because when you watched the television and then you hear on radio the education that is ongoing, it scared a lot of people, that is why they were not able to, you know convince the people and when they started the education and all those things people started scaring a lot of people and that if you are not very careful and they try it and then there is an outbreak then the whole community will suffer if not because of the outbreak unlike we all know that malaria the outbreak is there but we are not scared about malaria but the way *ebola* one came up and people were dying and maybe they said if you touch somebody that kind of thing even I myself was scared, it scared a lot of people about the whole issue and now that they want to try the thing on you, you know nobody yes so if there were not to be an outbreak but they know that there is Ebola oh but not on even a large scale whereby the whole world got to know about Ebola they were showing it, the way people were dying and those things ah the could have succeeded in making the trial.

Reference 2 - 3.40% Coverage

P: Yes the community members, so their reaction you see it wasn’t too bad but when the authorities came to start with this issue of why they are bringing this thing to my area you know the MP too they were making a little politics into it politics have entered into the whole so the MP want to say that as in a way of saving people that kind of thing in their area. So they started kicking against it I could remember this went to parliament and doctor Doodo was blasted and then all those things that he said something and he was asked to come and apologize to the house and all those things. Yes, you could see that actually, if not the higher authority trying to you know put fear into the community like this trial will have been successful, oh yes.

<Internals\\IDIs\\D-Opinion leaders-Hohoe\\S-IDI-64yr old Sub-Chief-Hohoe-05> - § 1 reference coded [4.52% Coverage]

Reference 1 - 4.52% Coverage

I: What are the factors influencing trust and the decision to participate in trial studies?

R: First of all is education, if you educate me well to know the benefits of it, then when I hear from a friend that it is good then I will also get involved

<Internals\\IDIs\\D-Opinion leaders-Hohoe\\U-IDI-female Public Health Nurse-Volta Region-07> - § 2 references coded [5.43% Coverage]

Reference 1 - 3.10% Coverage

Q: So what you are saying is that some people go behind to influence community members not to take part in the clinical trials and that is why trust is low in Ghana?

R: Yes

Reference 2 - 2.33% Coverage

Q: Would you say that was the reason why the ebola trial was not conducted in Ghana?

R: Exactly, exactly, that was what happened.

<Internals\\IDIs\\E-community members-Hohoe\\Y-IDI-49yr old male community member-Hohoe-04> - § 1 reference coded [5.75% Coverage]

Reference 1 - 5.75% Coverage

I: So do you know why the trial didn’t take part?

R: well from what I heard the chiefs and elders didn’t allow because of the rumors that were going on they stopped it. Some of the things I heard were that, people were saying the diseases had not gotten here yet so why did they want to do the trial so they refused because they said that could actually give them the ebola virus (IDI-49yr old male community member-Hohoe-04)

I: Please can you tell me some of the rumors?

R: Some of the things I heard were that, people were saying the diseases had not gotten here yet so why did they want to do the trial so they refused saying that could actually give them the ebola virus

<Internals\\IDIs\\F-MPs\\ZZ-IDI-54yr old MP-02> - § 1 reference coded [1.06% Coverage]

Reference 1 - 1.06% Coverage

And of cause what people read, for example Google is there and other things are there and they can easily Google and see what is it that you are going to do, and people even go to their pastors, laughter.

<Internals\\IDIs\\F-MPs\\ZZZ-IDI-59yr old MP-03> - § 1 reference coded [8.11% Coverage]

Reference 1 - 8.11% Coverage

R: As for religious beliefs, though people may say they are Catholics, Muslims and what have you, they still have beliefs in other things, now our society has become commercialized and so the most important thing is monetary value and one of the problems we face is that when we are trying to conduct these clinical trials like the ebola trial, those who opposed it were going round with fault hood information claiming that the people had brought plenty money and given to people who now want other people to be guinea picks (IDI-59yr old MP-03).

Q: Which people were saying that?

R: The Ghana Academy Arts and Science people were saying those things and they got people to be saying those things and even how much was given for that ebola trial. So if you look at that one alone is damaging enough for the trial. Even with the antimalarial they are recently giving, there was this girl who kept saying on WHATSAPP about the negative aspect of the exercise and saying if it were in Europe how much people were going to get as compared to here and with this kind of behavior it is be difficult for researchers.

**Name:** The issue of blood samples

<Internals\\IDIs\\A-Participated 3 or more times\\B-IDI-31yr old female-participated 3 or more times-02> - § 1 reference coded [2.77% Coverage]

Reference 1 - 2.77% Coverage

Q: What exactly do they say the blood is being used for?

R: They don’t say what they use it for, what they say is that they use it for their own things and not because of the health of the people and those people said because of that they will not want to be part of their work

<Internals\\IDIs\\A-Participated 3 or more times\\E-IDI-52yr old female-participated 3 or more times-05> - § 5 references coded [41.29% Coverage]

Reference 1 - 6.96% Coverage

R: In terms of the blood, some community members think that you (refers to researchers) take the blood samples to sell and make more money and that is what some people say. So such people believe that when they join they will take their blood and go and sell to white people and make money and that is why they would usually refuse to take part in such studies. For me, I don’t believe so, I think you take it and test to identify or know the disease that is in the child’s body.

Reference 2 - 10.41% Coverage

Q: Do people still have such perceptions in this community?

R: Yes, some still have that perceptions but it is not all of us. In my previous house, where I was staying before I came here, there was this woman we all gave birth at the same period, when they came to recruit us into the study, the husband refused with the reason that they can disturb, when they come they will ask you a lot of questions, they don’t give anything and they rather withdraw your blood and sell for money and he would not allow his child to take part. So he actually refused to allow the child to take part in that study. They (research team) came and spoke and yet the woman refused. So, such people do not trust the work you are doing.

Reference 3 - 9.09% Coverage

R: The issue is that at that time I was admitted with my child and all the expenses (hospital bills) I did were refunded to me by the study team and after they would collect the receipts from me. So each time they brought the refund to me, I was happy and when I told her, she would tell me that do I know how much they have sold my child’s blood? She would say I am only looking at the money they gave me and not looking at how much they have sold my child’s blood to the whites and collect money, it is only we the fools who will take part in their work. So if somebody has this perception, how will you convince the person?

Reference 4 - 6.77% Coverage

Q: Don’t you think community education will help address these perceptions?

R: Well, if that is done some will understand but other will not still understand. I have made them to know that for instance, if you fall sick and go to the hospital and they take your blood, do they go and sell it? And it is the same things the research people do. You will say all these to them and yet they don’t want to understand. So maybe you people can go and talk to them and see.

Reference 5 - 8.06% Coverage

Q: Would you say that there is lack of community opinion leaders’ involvement in our work and that is why some people have these perceptions at the community level?

R: Well, they have been talking to us at the clinics because there is no clinic that you will visit and you will not see research workers there. So there have been educating them but that is also their perception and what can you do. However, you need to continue to educate them and those who can change will change and those who will not understand, they should also have their opinion.

<Internals\\IDIs\\B-Refused and dropped outs\\F-IDI-28yr old female-Dropped out-01> - § 2 references coded [10.27% Coverage]

Reference 1 - 5.15% Coverage

R: They asked me and they said that one was not compulsory because if you want they will take but if you don’t want you can be with them without them taking the blood. But they said that whether the child is sick or not they will take the blood sample and I was like why would they take the blood if the child is not sick. For me if the child is sick that one, yes. So I even told them I am not interested in them taking the child’s blood and they also said they did not have a problem with that. So it does not have anything to do with the blood, I told them I am not interested to be part of their work again.

Reference 2 - 5.12% Coverage

R: I asked the one who administered the consent to me the reason why they take the blood and he was like they take the blood to go and test for what, and I said no, they should not take my child’s blood.

Q: Any reason?

R: I did not know the reason he was giving and that was why I refused. He said they would take the blood and test to know the sickness the child has and for them to treat it and I was like if the child is sick and you are taking the blood to go and check, I don’t have any problem with that but where the child is not sick and yet you want to take her blood, that one I will not agree.

<Internals\\IDIs\\B-Refused and dropped outs\\I-IDI-28yr old female-dropped out-04> - § 1 reference coded [2.84% Coverage]

Reference 1 - 2.84% Coverage

R: Exactly, I have, as for that one, you wouldn’t know what they are going to use it for, they just take it and they will not tell you they are going to test for this or that.

<Internals\\IDIs\\C-Opinion leaders-Nav\\M-IDI-47yr old youth leader-Nav-02> - § 1 reference coded [9.51% Coverage]

Reference 1 - 9.51% Coverage

R: Well, I can only talk about Navrongo and the issue is that when you are working in the Central and West, you don’t seem to have a problem but that of the East especially and South Zones, there are challenges. To take blood samples from a study child in the East Zone, then you have to be able to convince the parents otherwise they will tell you that in their house, they don t allow that. They would say in our tradition, we don’t take blood samples from small children who are two or three months old. So if you say that there is no way you can convince them again and so you just have to allow them otherwise you are looking for trouble.

<Internals\\IDIs\\C-Opinion leaders-Nav\\N-IDI-Paramount Chief-Nav-03> - § 3 references coded [4.00% Coverage]

Reference 1 - 1.23% Coverage

R: Yes, because at the beginning especially when they started the malaria trial you see most of the people have the perception that you know they used to take blood samples for trying most people refused to give out the blood for the trial. Because they have the mentality that it was not only for trying to get a vaccine for malaria but that they were using the blood for else, which they couldn’t explain.

Reference 2 - 2.17% Coverage

R: Yes, so majority of the people resisting to willingly let them take their blood sample so the research field workers found it difficult. They go to a house and they will be sacked that they are not prepared to be part of it, they are not prepared to give their blood samples out. But as time went on and the education was intensified I think now it is very simple. Any drug trial or any study that they want to do the moment they hear that research people are going to do a new study majority of the people or the communities are always prepared to join because they have seen the realities and the help the research has come to do so far as health is concerned. To my own observation I think now the level is high.

Reference 3 - 0.61% Coverage

R: If the education is not well done that is where you have such problems at other places because the level of understanding differs from community to community. So the problem is always the education.

<Internals\\IDIs\\D-Opinion leaders-Hohoe\\Q-IDI-59yr old male Assembly Member-Hohoe-03> - § 1 reference coded [0.57% Coverage]

Reference 1 - 0.57% Coverage

Q: And will that affect their participation if there is that kind of mistrust?

R: yes it will affect…it will affect seriously.

<Internals\\IDIs\\D-Opinion leaders-Hohoe\\T-IDI-male-Forma Director of health services-Volta Region-06> - § 2 references coded [6.21% Coverage]

Reference 1 - 4.68% Coverage

Q: What of the issue of blood samples taken during trial studies and community perception about that? Do you think it can affect the participation in the conduct of trial studies?

R: Yes, it does, even the trial we are doing now, we are taking blood samples and ideally, the blood should be taken in the evening and taking the blood in the evening the people really have some perception about taking blood in the evening and so we have to change it into morning they were taking that taking blood samples in the night was not right. You know blood too, the way people look at blood, once you are taking blood, you matter how small or the quantity you are taking you know people are apprehensive about it and so you need to explain things to them (people) and for them to really understand what you are going to use the blood for and all that but blood is also an issue

Reference 2 - 1.53% Coverage

Q: So then am sure if you people had not listen to the people to change the time you use to take the blood from evening to morning, am sure people would have refused to be part of your study?

R: Yes and even am sure some people may even drop from the study because of the blood issue

<Internals\\IDIs\\F-MPs\\ZZ-IDI-54yr old MP-02> - § 1 reference coded [4.17% Coverage]

Reference 1 - 4.17% Coverage

They should not use the samples for any other purposes apart from what they said they were going to use it for. That is the fear of some patients and I remember em there were times when people were coming to donate blood and if you did not council them properly and you took a blood sample and went and tested and you now wanted to bring some information back to the patient and to tell the patient that this thing has been found in your blood, it became a problem because you did not explain to me that you were going to test for Hypetisis B, HIV or anything like that and you are now coming to give the patient this information. So I think for researchers, they should keep to their words and use samples they take from their clients the purpose in which they tell people they are going to use it for.

<Internals\\IDIs\\F-MPs\\ZZZZ-IDI-49yr old MP-04> - § 1 reference coded [4.01% Coverage]

Reference 1 - 4.01% Coverage

Q: Some people have the perception that researchers take the samples and sell. Do you think that is the case?

R: I don’t share in that perception though it does exist and that is why earlier on I said sometimes is the way we explain these things to our people knowing very well our background in terms of education, we still have a very large percentage of people not educated and so their level of understanding especially scientific research is not that great and so sometimes *eeem* because of that and socio-cultural background, the fear is there that oh you are going to use it for juju or some superstitious beliefs and things, you know in our environment we have those kinds of beliefs. Therefore, if you are taking blood or such samples from somebody and you don’t explain then he will say oh no he’s taking my blood for other superstitious beliefs, which really for me, I don’t share that.

**Name:** uncertainty of treatment effectiveness

<Internals\\IDIs\\B-Refused and dropped outs\\I-IDI-28yr old female-dropped out-04> - § 1 reference coded [4.36% Coverage]

Reference 1 - 4.36% Coverage

R: Yeah, it not actually from me, it was my husband who refused and his worry was that the thing (refers to the malaria vaccine) is new and there are still piloting it and that is the reason why he has refused for the child to take the malaria vaccine and nothing more.

<Internals\\IDIs\\C-Opinion leaders-Nav\\L-IDI-53yr old male Assembly Member-Nav-01> - § 1 reference coded [6.08% Coverage]

Reference 1 - 6.08% Coverage

Q: What are the factors that can affect trust and participation of people in trial studies?

R: One of the factors that can affect trust is religious beliefs. In some of the churches, members do not belief in *eeeh*, what do we call it, modern drugs, what they do is that they don’t need to take drugs, they don’t belief in drugs and they belief that whoever is sick is the will of God. So there are some of the churches that are like that and that can affect your work because then they will not take part. The other thing is also that the lower educated people in the communities have the believe that some of the drugs are really not going to help them, even some of them say that the drugs will lead to impotency while others think some of the drugs when you take them it reduces the number of years you are going to live on earth. These beliefs are coming from the very remote communities and not my community where people are enlighten. The last thing I would want to talk about is government policy, you know the ebola trial ended up in Parliament and some of the things that will happen and will not fetch votes for a particular party in Government, they will not be willing to push such an agenda. So, political factor is also one thing that can affect some of these things.

<Internals\\IDIs\\F-MPs\\ZZZZ-IDI-49yr old MP-04> - § 1 reference coded [3.66% Coverage]

Reference 1 - 3.66% Coverage

Q: What of uncertainty, side effects and capacity of trial team, do you think these also influence trust of people negatively of positively?

R: You mean the drugs will work or is good for them or?

Q: Yes and may be the side effects. Progressing

R: Yes they are also factors that can influence trust especially the side effects, once they are not very certain of what they are taking, *aaah*, the fear is there and because the fear is there, the trust is low because the person is so afraid that ooo this thing will rather lead him/her to other conditions or harm him/her and that is where the issue of trust begins and if I’m not sure of what this drug would do then I don’t trust it. So, I agree that uncertainty about the kind of drug they are taking would definitely affect trust and participation, yes.

**Name:** use as guinea pigs

<Internals\\IDIs\\D-Opinion leaders-Hohoe\\T-IDI-male-Forma Director of health services-Volta Region-06> - § 1 reference coded [3.67% Coverage]

Reference 1 - 3.67% Coverage

R: The problem is that there were some people who were actually against it (refers to the ebola trial). There are some people who are of the view that the whites usually come and use us as guinea pigs you know to conduct these trials and these people had a bigger voice than the researchers at that time and because our people too are not well informed about these things (trials) they also quickly believed them. So information about the trial, those who were actually against the trial took the lead and informed the people about the bad side of the trial and before the Scientists could come in those guys (people) have done a lot of harm already *ehaa* and that was what happened.

**Key Informant Interviews (KIIs)**

**Name:** Experience in previous trials

<Internals\\KIIs\\A-Clinical trials researchers\\E-KII-48 yr old clinical trial Monitor-05> - § 3 references coded [8.01% Coverage]

Reference 1 - 3.94% Coverage

Q: Are there other factors that you think influence trust negatively and participation of people in trial studies?

R: Yes and I think it depends on previous clinical trials conducted in the area and what happened you know, and that one can influence trust because if what they expected and what they were told, they did not see that when you come again and you are saying it, they will not believe you. So really, it will depend more on previous trials conducted, how the research team conducted themselves because that is where the issue of trust comes from. Apart from that what people have heard on radio or seen on TV concerning some trials conducted somewhere else all can inform or influence trust when it comes to the conduct of clinical trial studies.

Reference 2 - 2.48% Coverage

Q: What of the issues of compensation and honesty on the part of trial team members?

R: Oh yes and so depending on previous trials and how everybody conducted themselves concerning what they will give may be during the consenting process, for instance when you come we will give you refreshment, pay your transport and all that and if they are not seeing or getting that when they take part in the study can all affect their trust in future studies to be conducted in the area.

Reference 3 - 1.59% Coverage

Q: What of the issue of side effects and doubts about the effectiveness of the new medicine to be used?

R: It all depends again on previous trial studies and as I said of it did not go on well to their expectations regarding these factors you have mentioned, when you come again then they will be reluctant.

Internals\\KIIs\\B-Ethics members\\I-KII-42yr old female ethics committee member-GHS-03> - § 2 references coded [11.91% Coverage]

Reference 1 - 9.34% Coverage

R: It is all about the education, you know if you are not able to explain your information sheet well to the people (not able to administer informed consent well), if you are not able to do your community entry very well, if you are not able to explain the issues in the language that the person understand to enable them take informed decision then that is where the problem is. So even with the normal protocol, if you are not able to explain the issues for the person to know whether he/she should take part or not then that is where the problem. So the information sheet is very important and it depend on how simple it is and how people are trained to explain the study procedures to the participants to really understand and that is what is going to build the trust. So for instance if you give the information sheet to the person to take home and discuss with another person to probably build on his/her interest then depending on the person’s view on that thing (refers to the study) and what the person has heard before, if there is so many misinformation about it, that is where they might not be trust or something has happened in the community before and they did not understand it well and that is still in their minds and when you go there with a new study, based on previous experience then that is where the trust might not be there.

**Name:** inadequate informed consenting procedures

<Internals\\KIIs\\A-Clinical trials researchers\\A-KII-53yr old clinical trial investigator-01> - § 3 references coded [9.70% Coverage]

Reference 1 - 4.35% Coverage

**Use text**

Q: What of informed consent, does it affect trust and participation in trial studies especially when it is not done well by study team members?

R: That is very true and very central when it comes to clinical trials especially the long term trials such as 4 or 5 year trials. What keeps participants in the study is the informed consent, if they are told exactly what is involve in the study. Sometimes, if your informed consent is badly done then the people don’t know what to expect so sometimes you go and they say oh we thought you have finished with us. Informed consent makes them feel respected because they ask questions and you take your time to answer them, then you also ask them whether they have further question, I can go and come if you want to think about it or something and they will say oh you stay it is ok. It makes them feel important and so when they take part they know that they have voluntarily taken part and not that they are being push. If you force them may be in front of you they may not do anything but when they go back home they will not come back.

Reference 2 - 2.50% Coverage

Q: Though clinical trial researchers normally train their fieldworkers but do you think they usually do the right thing when it comes to informed consent administration?

R: To the best of my knowledge, they do the correct thing and any fieldworker who is found not to have done the correct thing, the person is dealt with and that particular consent is re-done. The informed consent is very vital in clinical trial studies and the ethics emphasizes that the right thing should be done and if it means you go and come back and do it you have to do that. Where we find inconsistent behavior we have to deal with that fieldworker.

Reference 3 - 2.85% Coverage

**Use text (informed consent)**

Q: But would you say other places where may be it is not done well then it can affect participation?

R: It should not be a problem, yes. Anywhere informed consent is not done well it may be a problem because if fieldworkers go and tell people that oh whenever you are sick, we will pay everything for you and if that study is not doing that and for them to get participants they will tell them these things and if the person is sick and does not get that care then they get offence and go aware. If you do that and the people think that you are coming to deceive them it may be a problem for you because it contribute to lack of respect for the people and that can jeopardize the study.

<Internals\\KIIs\\A-Clinical trials researchers\\B-KII-48yr old trial coordinator-02> - § 1 reference coded [5.03% Coverage]

Reference 1 - 5.03% Coverage

Q: So then would you say informed consent is a problem and we don’t do it right as ethics required?

R: *Oooh*, as for that one, don’t go there my brother because it is a huge problem. As for informed consent, I don’t know whether we should call it informed again. Sometimes, we even translate these consent forms into the local language but when we go into the communities, we give the English version to the people and only send the translated one to ethics and how many people at the community level can read and write? Even those who can read, you are supposed to give the information sheet to them to read and after they have read, they are supposed to ask you questions for you to answer concerning all their fears and concerns. But what we do is that you know we are from so, so and so place and we are conducting this study….so we will give them the information meanwhile we have not told them everything they need to know and then ask do you have a question? That short time, the person will say I don’t have any question then for the person to come the next time and say I don’t want to be part of it because now I have read the thing and got the details and so I don’t want to be part, So informed consent is a huge problem for us and I don’t know whether it has to do with the training we give to research officers or data collectors. I think we overlook informed consent and we don’t pay much attention to it and that is causing some of these problems and we need to pay much attention to it and do it well.

<Internals\\KIIs\\A-Clinical trials researchers\\E-KII-48 yr old clinical trial Monitor-05> - § 1 reference coded [3.39% Coverage]

Reference 1 - 3.39% Coverage

Q: Do you think education in that regard can influence their trust positively for them to get involve in trial studies that take blood samples.

R: Yes, consenting process has to be done very well and not just telling people what the study is all about but you know going outside to let them know why the trial is being conducted, the importance of it and then they contributing their time in order to allow the trial to be done so that we will be able to find drugs or vaccines to help humanity. So education and on the need to conduct these trial studies and then consenting process should be done very well in order to build trust of community members.

<Internals\\KIIs\\A-Clinical trials researchers\\F-KII-38yr old clinical trials monitor-06> - § 1 reference coded [2.95% Coverage]

Reference 1 - 2.95% Coverage

Q: Would you also say the way we informed consent is administered by trial team members during trial studies is something that affects trust and participation?

R: It could be a problem but that should not be a problem for clinical trial investigators because they are very experienced people and they know what to do but the problem however, has to do with over side. So then you have to do supervision, you have to ensure that the principles that are laid down for the conduct of clinical trials are followed. You have to do routine supervision, spot checks and all that so that the people (field staff) don’t relax at any point in time you understand? But they (refers to trial investigators) turn to get busy and they don’t do some of the things they are supposed to do, yeah.

<Internals\\KIIs\\A-Clinical trials researchers\\G-KII-55yr old clinical trial investigator-07> - § 1 reference coded [4.14% Coverage]

Reference 1 - 4.14% Coverage

Q: Would you say poor informed consent affect trust and participation in trial studies?

R: The issue is not about poor informed consent though informed consent is absolutely important and people should always do it well. Informed consenting is at the individual level, however, in conducting clinical trial one need to understand the context and cultural dynamics of the place and try to address that and that is why informed consent is not just with an individual but it got to be a process, a process of engagement, a process of getting feedback, a process of being able to change and then a process of actually signing the consent form, and so the consent form is just one aspect of the whole process.

<Internals\\KIIs\\B-Ethics members\\G-KII-54yr old male ethics Ccommitteemember-Nav-01> - § 1 reference coded [3.65% Coverage]

Reference 1 - 3.65% Coverage

Q: Would you say that informed consenting also influences trust of people when it comes to their decision to take part in trial studies especially if the trial staff is not able to explain the issues very well to the person?

R: Yes it influences somebody’s decision to take part and that is why when you go to the community, you have to get somebody to translate into their language to understand. So the consent form should be such that it should be able to influence or let the person accept that fact that what you are doing is important and for him/her to be part.

<Internals\\KIIs\\B-Ethics members\\J-KII-59yr old male ethics committee member-GHS-04> - § 2 references coded [5.15% Coverage]

Reference 1 - 2.26% Coverage

R: I rather think that informed concerned gives trust because this one that you have left for me and if I am even uneducated and I put my thumbprint there, I know where to contact you. So informed consent gives evidence that these people can be found somewhere and that for me builds trust of people and that is why we (refers to ethics committees) insist that you give a copy of the concerned form to the participants. Sometimes when we go for auditing at the community level we ask people whether they were given consent forms and when they show it to us we ask them to keep it very well. So I think it increases trust rather.

Reference 2 - 2.89% Coverage

Q: But if you look at it in terms of the fieldworker’s ability to explain the issues very well to study participants to understand and then take a decision whether or not to take part in the study. Where trial fieldworkers are not trained well to be able to administer the informed consent very well, don’t you think that it negatively affects trust and participation?

R: Yes for that one, it can affect it negatively and that is why sometimes the brochures that come with these things, they have pictures because pictorial is one of them and you sometimes need to show these picture to the people and that will improve understanding. However, what you are saying is very true that if the fieldworker cannot explain the issues very well it is difficult for people to buy into what you are going to do.

<Internals\\KIIs\\B-Ethics members\\K-KII-51yr old female ethics committee member-Nav-05> - § 1 reference coded [2.76% Coverage]

Reference 1 - 2.76% Coverage

Q: Then based on what you are saying it means some fieldworkers sometimes tell study participants things that are not in the consent form?

R: Well I am not there to see, so I can’t say they say their own things. Sometimes the participants are looking at how they will benefit and also based on what previous studies have done they assumed that this current study will also do the same thing for them. However if we identify such community people with such concerns, we will go back and tell them that we do what the consent form says and we don’t go out to do things that are not in the consent form of the study.

**Name:** lack of education & engagment

<Internals\\KIIs\\A-Clinical trials researchers\\A-KII-53yr old clinical trial investigator-01> - § 1 reference coded [2.00% Coverage]

Reference 1 - 2.00% Coverage

Q: Would you say stakeholder engagement was not properly done or?

R: It is not that stakeholder engagement was not properly done, it was not fully done. The issue is that the company comes to engage the local researchers and that is the first step, then the local researchers will engage Ministry of health, Ghana health service and those things were done because they were all in the picture. What is left is to now engage the traditional rulers and the community, which you can’t do without approval.

<Internals\\KIIs\\A-Clinical trials researchers\\B-KII-48yr old trial coordinator-02> - § 1 reference coded [4.51% Coverage]

Reference 1 - 4.51% Coverage

Q: What if the level of trust when it comes to the conduct of clinical trials studies in Ghana?

R: I think on a scale of 1 to 10, the level of trust I will say it is different at different levels, on the scale of 1 to 10 and among the scientists, I will say the level of trust is up there like 8. When it comes to within the health system, the level of trust will be 6 and for then at the community level it is almost like about 2. For the media I think the level of trust for clinical trials is almost nothing (no trust at all).

I think the reason for lack of trust in the conduct of trial studies is because of disengagement. It is because Scientists don’t engage much and if people don’t know why, they will ask why, you understand? If I don’t know why you are doing a particular thing, I will ask you why? And because we are not engaging more, we leave a very big loophole in there and for that reason, the media will start juking (will pick it up) and say they are hiding, they are hiding, it is not true, they say this vaccine will do this and that, it is not true and if not why are they not coming out clearly? You see it? Then the politicians will also have their own level you know to score political point (KII-48yr old trial coordinator-02).

For me, I think most Scientists understand the importance of clinical trials because without clinical trials, we cannot be where we are today in terms of health.

<Internals\\KIIs\\A-Clinical trials researchers\\C-KII-58yr old clinical trial investigator and Director-03> - § 1 reference coded [6.57% Coverage]

Reference 1 - 6.57% Coverage

R: What are the factors that influence trust of people and a decision for then to get involved in trial studies first at the community level?

R: At the community level, they have the hierarchy the traditional hierarchy and so they have the Chiefs, they have the elders and the opinion leaders and then they have the participants themselves. When you go and see a participant he/she may want to go and discuss it with the chief and say oh this is what these people have come to say should I go ahead and take part or not and if that Chief tells him that oh, they have spoken to us, we think it is clear and so there is nothing to be afraid of but it is just your choice now to be part of not then it carries a heavy weight. However, if the person goes to the chief and the chief tells him that oh they came to talk to me but this thing are suspicious definitely the participant will not take part. So to be able to build trust at the community level, it is good to start with the opinion leaders, get them to understand and give them the opportunity to ask all their questions and truthfully give them the answers and then you organize the durbars where everybody comes to listen and ask their question. Then when it comes to them one on one, it is nothing new to them. So that trust is important at that level. The other thing is the past experiences, if they have worked with the people before and they realize that these people who came and told us that they would do this for us they actually did it and so that trust is there and when you come again in future to do something with them then they know these are people I can trust. If they don’t know you and they are skeptical they will say these ones we don’t know them and what if you come and harm us then is a problem. So building the trust is also very important.

<Internals\\KIIs\\A-Clinical trials researchers\\D-KII-50yr old clinical trial investigator-04> - § 1 reference coded [3.19% Coverage]

Reference 1 - 3.19% Coverage

R: You see communicating it in the local language in itself is a problem. Twi is the most viable language in Ghana and how can you translate clinical trials in Twi? For me as, I cannot think of how that can be translated in Twi and that is where the difficulty is but I think that should not be am excuse for us not to do that. So we need to involve school of languages to help us see how we can translate these things into the local language to help us educate people about trial studies (D-KII-50yr old clinical trial investigator-04).

<Internals\\KIIs\\A-Clinical trials researchers\\F-KII-38yr old clinical trials monitor-06> - § 2 references coded [7.18% Coverage]

Reference 1 - 2.84% Coverage

R: The problem is that, majority of Ghanaians do not even know what clinical trials are all about and that is the underlining fact. So, before they can even trust clinical trials, you have to look at what is the awareness level, how many of them actually know what a clinical trial is? In fact when they are talking about research, people think it is just laboratory and when you are now involving human beings then they say *ah* why, you want to use us as rats or guinea pigs? (KII-38yr old clinical trials monitor-06)

And so for me, the people who are directly involved (refers to the study participants) in the conduct of clinical trials, I can say trust is high because they are getting firsthand information on what they are getting (refers to benefits) and so they will trust the process.

Reference 2 - 4.35% Coverage

However, at the national level, people who are at a distance will have different perception about clinical trial studies and when they think about clinical trials they only remember the bad cases of clinical trials and not the good cases. If somebody is having headache and goes to take paracetamol, Paracetamol has gone through clinical trials before, when you have malaria, you quickly run to take coartem, coartem was once a clinical trial product, *heee*, you see and so they are looking at, they don’t know how those drugs came about. They are quick to remember the bad aspect of clinical trials and for me, it is not about *tey,* they don’t trust but they don’t have the information, the knowledge. So the ignorance is making us to think they don’t trust but if those people, the front liners opposing clinical trials, if there is a situation, they will trust clinical trials. For example, let any of those people (refers to clinical trial opposition) have a terminal disease such as cancer and we say okay, we have a trial product, which has the potential to help you, they will quickly go for it.

<Internals\\KIIs\\A-Clinical trials researchers\\G-KII-55yr old clinical trial investigator-07> - § 3 references coded [10.96% Coverage]

Reference 1 - 6.10% Coverage

Q: Ghana Academy of Arts and Science follows were also against the ebola vaccine trial. What was their main issue?

R: Well, their issues were in line with the issues other people had and so they thought that this was a vaccines that was being tested to prevent the disease and therefore they thought that we were going to bring in the disease with the vaccine to test and see whether the vaccine works or not but who will do that? So the lessons we have learned is that even with the highest level of education people are still very well informed and I think there should be much efforts to inform them about the conduct of clinical trials. So a political disease like ebola and for Ghana academy and sciences to say the kind of things that happened meant that there was lack of communication. The Ghana academy people, who are they? They are made up of professions and doctors from different backgrounds but their knowledge of this particular issue and their information was lacking and also the current methods of vaccine development.

Reference 2 - 2.64% Coverage

So the lessons I have learned from this issue is that we don’t take anything for granted. A mathematician who is a member of Ghana Academy is a mathematician, a Historian is a Historian *eem* an epidemiologist is an epidemiologist and so we don’t have to take things for granted that *eem* an epidemiologist who has done epidemiology about diseases should therefore know about the conduct of clinical trials. So, that engagement needed to have been done.

Reference 3 - 2.22% Coverage

Q: To educate them about the trial?

R: Yes but you see the other thing is that even if that was not done they it was important for them to also take a bit of time to get that information and understand the trial before putting out that information because if you get information from Ghana academy people and from such reputable people, which we all respect you will accept it.

<Internals\\KIIs\\B-Ethics members\\G-KII-54yr old male ethics Ccommitteemember-Nav-01> - § 1 reference coded [4.35% Coverage]

Reference 1 - 4.35% Coverage

Q: People were saying we did not have the here and yet they are going to conduct such a trial. Some people believed that conducting the trial was going to introduce the ebola disease into the country….?

R: Okay, so that is the education I’m talking about, if you are educated as to how those things (refers to trial studies) are done then, you wouldn’t go around and say conducting the trial would bring the ebola virus here because there is a way those things (refers to trial studies) are done. So if you come and you educate me and I understand it, I wouldn’t go and stand somewhere and say *eeeh*, *eeeh* what is not true, yeah. So the education was not properly done in my view.

<Internals\\KIIs\\B-Ethics members\\H-KII-47yr old male ethics committee member-Nav-02> - § 2 references coded [6.68% Coverage]

Reference 1 - 3.36% Coverage

Q: but whether trial or whatever I mean that kind of, do you think there is something that we all didn’t do right and probably that is bringing those issues?

R: for the recent example, like the national publicity was not done well especially the radios… you know delayed and short time they brought funds and then they are going ahead you see when the trial itself was done long time ago and the people might have forgotten so if you come and select few communities or few districts that you are doing this they will surely they have knowledge about what has been happening here so they will say it is a trial so the public education was not done very well so that it will remind people that even if sample were taken in this area they will know that it has already been done now we are only trying sorry, piloting so that we document few points and then extrapolate it to the general population .

Reference 2 - 3.32% Coverage

R: The factors, the part that they did not do right is maybe informing the public very well and especially in the midst of what is called Ebola some people know it is a trial, was it not a vaccine they were trying? Some believed that it was a weaken Ebola virus that is going to be used like a weakened attenuated vaccine, it may even introduce ebola into the system some were very much afraid. Those who are knowledgeable convinced those who are not aware of it and besides that if they were very cautious and informed the media even met the media about the whole nitty-gritty about the whole matter about how they will do it and convinced them very well and they understood it, they wouldn’t have bloated the whole thing as it happened because they, whatever they say the public get it right so when they are also not convinced they are afraid it also instigated the public not to go ahead.

<Internals\\KIIs\\B-Ethics members\\I-KII-42yr old female ethics committee member-GHS-03> - § 1 reference coded [2.63% Coverage]

Reference 1 - 2.63% Coverage

Q: What do you think was not done right and that made the ebola trial not to come on in Ghana?

R: First and firmost they did not do the community entry well, the community they were going to engage to be part of the research, they did not engage them well. Enough information was not given and for the people to decide whether they would be part of the study or not. There was inadequate information and that made people to also come up with their own version of information about the ebola study.

**Name:** risk-fear & harm of new drugs

<Internals\\KIIs\\A-Clinical trials researchers\\C-KII-58yr old clinical trial investigator and Director-03> - § 1 reference coded [2.92% Coverage]

Reference 1 - 2.92% Coverage

The sad part of it came from the Ghana Academy of Arts and Sciences who are supposed to be very senior scientific people in the field and if they themselves were also raising questions then it got everybody quite scared and alarmed that *eeh*, something is really going wrong and so those were the issues that brought about the mistrust and I think lessons have been learned and going forward I think we have tried to address them. The ebola vaccine was suspended because the cry was so much; I mean it was coming from so many sectors but then later on when parliament gave the go ahead for the trial to be conducted the sponsor had already gone and it was an unfortunate situation where Ghana lost that opportunity to carry out that exercise to help us in case the outbreak comes in.

<Internals\\KIIs\\A-Clinical trials researchers\\E-KII-48 yr old clinical trial Monitor-05> - § 2 references coded [6.38% Coverage]

Reference 1 - 2.88% Coverage

R: I would not say the trail team did not do well because they did what we normally do in Ghana before such studies are conducted. The ebola trial had its own issues that generated what happened because if you just hear ebola, you start running, everybody start running and so that was what caused the issue and for it to be taken to Parliament. When people hear about ebola then their blood pressure start going up, laughter (KII-48 yr old clinical trial Monitor-05)

When people hear about ebola then their blood pressure start going up, laughter, otherwise we have been conducting clinical trial in Ghana not today util the ebola issue came, which is a total different issue.

Reference 2 - 3.50% Coverage

Use this code

I can tell you for instance in Gambia, when the president heard that MRC (Medical Research Centre) in Banju, Gambia wanted to conduct the ebola clinical trial, the president I can tell you summoned the director of the institution because of that. Meanwhile they have also been doing clinical trials in that country for years and so the ebola thing is a different ball game (issue). The President had even closed his country’s boarders and then he hears somebody wants to conduct ebola trial in his country without his knowledge and so he asked then to stop. So the ebola trial is a different issue and one should not take a decision using the ebola trial issue.

<Internals\\KIIs\\B-Ethics members\\H-KII-47yr old male ethics committee member-Nav-02> - § 1 reference coded [3.45% Coverage]

Reference 1 - 3.45% Coverage

R: per my little experience here especially the malaria vaccine that we are introducing, the comments from some people here some even refused it that we are doing a trial instead of piloting you say we are doing a trial and so some refused to be part of it. And their reason is that they don’t know what the vaccine will do to their children. When we met the assembly people some of them were supporting the village people that since it is a trial, they have asked their wives or whatever to refuse it (47yr old male ethics committee member-Nav-02)

So it means that here because research is here and is been on the people on several occasions some people have become more concerned about what the frequent trials, I mean trial or whatever we call it that we usually the population here to do may have on them so it affects certain work that we even do which are not especially this one was a piloting but they said it was a trial so personally I know that they refused it when we met the assembly people some of them were supporting the village people that since it is a trial, they have asked their wives or whatever to refuse it.

<Internals\\KIIs\\C-FDA members\\M-KII-38yr old male-FDA-02> - § 1 reference coded [9.87% Coverage]

Reference 1 - 9.87% Coverage

R: The parliamentarians were more or less influenced by the Ghana Academy and Science people and they taught that they are also a group of people who are educated and in any case, FDA is also a science institution that looks at the science before approval. However, their argument was that look ebola os not here and why are we going to do the ebola trial here and I think *eem* with your basic science and somebody in elementary school should be able to dispute that. The reason is that it is a vaccine and you do not introduce vaccine in disease population, you give vaccines to people to prevent them from getting the disease and so this was a basic thing but you see the disease itself, the mention of the ebola makes people to panic and sometimes it can make you to forget your basic science, laughter. The other thing was that they were concerned that the conduct of the trial in Ghana, wouldn’t it cause the disease to actually come. You see the academy people had not seen the protocol because it comes to the FDA and if really they needed anything they should have come to the FDA to ask questions bit they doid not come. If you look at the ebola trial it was a weaken virus, atuanated one that they wanted to use which was very safe for participants and there was no way participants were going to get the ebola virus as some of the people claimed. It was one of the safest vaccine that was being used, which people did not understand but after they made the noise and we had to actually brief the minister of health, we also went to parliament and presented the issue and they all realized it was not the way they were thinking and they actually said ok we could go back and conduct the trial but then the sponsor had withdrew.

**Name:** The issue of blodd samples

<Internals\\KIIs\\A-Clinical trials researchers\\A-KII-53yr old clinical trial investigator-01> - § 1 reference coded [4.14% Coverage]

Reference 1 - 4.14% Coverage

R: Drawing of blood has been a very big problem and whenever blood is going to be drawn its really makes research or clinical trial very difficult because you have to go the extra mile to explain things to them and to demonstrate to them that you are trying to take a minimal volume of blood. The reason is that blood is very significant in our local communities because some of them believe that life is in the blood and that is true and so if you take somebody’s blood you have actually come in contact with the person’s spirit and so they are very particular about it. Even when you explain and they have understood, any wrong move on your part, they begin to mistrust again. Dealing with blood in our research work, you need to be very careful because if one person suspects something it brings out mistrust and there will be Rumor that the blood is going to be sold. But here in Navrongo we have demonstrated transparency in blood draw and things like that in several communities. So for blood draw studies, you must be very careful.

<Internals\\KIIs\\A-Clinical trials researchers\\C-KII-58yr old clinical trial investigator and Director-03> - § 1 reference coded [4.87% Coverage]

Reference 1 - 4.87% Coverage

R: Yes I have heard of some of them but *eeem* not really had personal experience of participants complaining to me but I have heard of such complains and of course culturally, blood is life and so the people identify with their blood, they are very careful who is taking it and what it is being used for. Also, culturally, you know that you can use people blood for some harmful practices and so they are skeptical if you are taking their blood. Lastly, there is also a rumor that the blood is being sold and why should they (researchers) be taking from them for free and so they turn to be apprehensive of the blood but one study we did where we took blood samples for malaria study and the blood was going to be stored for a while and so the people had agreed for the blood too be taken and stored and even after 2 years, one of them came and was asking so is his blood still safe where it has been pot, laughter and so they attach some feeling to the blood and I would have thought that after 2 years they would have forgotten about it but no they still have issues with the blood and whether it been kept and whether it is still safe or not. So blood is very important to individuals and once it has to be taken, it has to be very well explained to them why we are taking it and what it is to be used for.

<Internals\\KIIs\\A-Clinical trials researchers\\E-KII-48 yr old clinical trial Monitor-05> - § 1 reference coded [6.44% Coverage]

Reference 1 - 6.44% Coverage

Q: When it comes to the issue of blood samples, there are various views being expressed about it by some people. Do you think the issue of taking blood sample has any influence on trust and the decision for people to get involve in trial studies?

R: Yes it does because clinical trial studies that involve a lot of blood samples you hardly find people consenting to take part. People have various reasons and sometimes it is not perception nut it id about the pain and the person thinks that he/she is going to bear the pain with the finger print and he cannot enjoy the pain and so it is not always perceptions but the an aspect of pain that they have to bear. Then also the person will consider the number of times he/she has to come for them to take the blood samples and if the person will not have time because he is busy with his work and he has to leave his work, leave his family to come to the hospital or bringing the child the hospital for the sample to be taken *ehaa*, that inconvenience is there and those are the factors and not perception. Also, in some areas or village setting where they think that you are taking their blood for something else and not scientific then it affects their willingness to take part in the exercise.

<Internals\\KIIs\\B-Ethics members\\G-KII-54yr old male ethics Ccommitteemember-Nav-01> - § 1 reference coded [8.95% Coverage]

Reference 1 - 8.95% Coverage

R: Well, individuals may have their own perceptions but you know, *eeeh* you give the scientists the benefit of the doubt because you have come to say you want to use this sample for this specific purpose and then you then go and use it for something else, you know there are legal issues backing people who give ethical clearance. So if you go and use it for different thing, nobody may catch you, but when you are caught, then the law will deal with you. If the researcher comes and submits the protocol and says I will use the sample for this purpose and you (ethics members) say I have this perception that you might go and use the samples for a different purpose and so I will not give it to you (approve the protocol) then eeeeh it comes to a standstill, the research cannot be done. So as am saying, you put so many things in place that will check the researcher because it is not only one person who does research, it is a collaboration. Mr. A is coming from South Africa, Mr. B is in Ghana and they say they want to do this particular research and they need this particular sample, *eeeem*, we will trust them to some extend then you pray that they use it for what they say they want to use it (samples) for. Yeah, those perceptions are there but since time memorial, I don’t think anybody has been caught using blood samples for other purposes rather than what they intend using it for, yeah.

<Internals\\KIIs\\B-Ethics members\\J-KII-59yr old male ethics committee member-GHS-04> - § 1 reference coded [3.59% Coverage]

Reference 1 - 3.59% Coverage

R: Yes, it (refers to cultural and religious beliefs) will because if you are taking samples like blood and you go to a place where they have Jehovah Witness and that is why it is good to have this community engagement to help you know the profile of the community. I think in the past we played down on these social things but now it is very critical because if you know you are going to for instance Muslims community and you cannot get males to examine women, then you must know how to play your cards (how to handle things). So those are critical issues that one has to consider when doing some of these trial studies I some selected areas because one thing in one selected area will be successful because of their correct mix (refers to mix of both female and male field staff) and the other thing is if you go there with a wrong mix then it may not work well form you. So the cultural and social norms are important and must be taken into consideration when you are conducting trial studies.

<Internals\\KIIs\\B-Ethics members\\K-KII-51yr old female ethics committee member-Nav-05> - § 1 reference coded [1.43% Coverage]

Reference 1 - 1.43% Coverage

Q: Some community members said that researchers take blood samples from participants and sell or use the samples for different purposes. What is you view on that?

R: Well that one, I think is a cultural perception that has been there for ages, so you just have to continue to talk to them and tell them it is not so (KII-51yr old female ethics committee member-Nav-05).

<Internals\\KIIs\\C-FDA members\\M-KII-38yr old male-FDA-02> - § 1 reference coded [2.15% Coverage]

Reference 1 - 2.15% Coverage

Q; But community members still have concerns when it comes to the issue of blood taking. Has that come to your notice?

R: No it has not come to our notice but if they think that is an issue, they should not sign and be part of it. However, it is the duty of the investigator to explain to them that they are using the blood samples for the purpose in which they have taken them.

**Name:** the issue of rumor and bad influence & wrong information

<Internals\\KIIs\\A-Clinical trials researchers\\A-KII-53yr old clinical trial investigator-01> - § 2 references coded [8.78% Coverage]

Reference 1 - 3.60% Coverage

R: Those names are known in Navringo and the trust is there in Navrongo for such names but out there in Volta Region I don’t know whether they did not trust their CVs but the rumor started first in the Volta Region. If it had been in Navrongo, even if the rumor comes here the people would have come to find out from us first and they wouldn’t have believed it. The names may be there but the ebola trial was like the first of its kind in that area. You see we have some roaming researchers who come and they take blood samples and there are gone unlike us here we are staying with them and even if the research finishes, you are with the people and if you do something wrong they will come to you in the office or even your house to ask questions. So even if you are coming with a very good idea, you have to pass through those of us who are here because they trust that we will always do the right thing.

Reference 2 - 5.17% Coverage

**Use text**

Q: What other factors would you say affected the conduct of the ebola trial in Ghana? May be on the part of the researchers?

R: One thing is that if you get ethical approval to conduct a trial study and you go to the community, there is a lot that goes into research. Lone before the research starts, you can negotiate for a trial for 2 years and you are talking up and down. Within these 2 years the conception is there and everybody knows what you are trying to do but without ethics giving approval, you can’t go and tell the community people that you are coming to conduct a study. So, if a bad person just picks it up and goes to the community there is nothing you can do because you cannot go to the community before that person and say no this is what you are coming to do because you don’t have the permission and that is one of the principles. So that was what happened with the ebola vaccine trial that a few scientists knew what was going to happen and they went ahead to the community to give them wrong information and there is nothing the researchers could done once you don’t have ethics approval, you cannot go and tell the people that this is what you are coming to do. So a bad message was sent to the people and there was nothing the researchers could have done without ethics approval (KII-53yr old clinical trial investigator-01)

<Internals\\KIIs\\A-Clinical trials researchers\\B-KII-48yr old trial coordinator-02> - § 2 references coded [9.15% Coverage]

Reference 1 - 4.51% Coverage

Q: What if the level of trust when it comes to the conduct of clinical trials studies in Ghana?

R: I think on a scale of 1 to 10, the level of trust I will say it is different at different levels, on the scale of 1 to 10 and among the scientists, I will say the level of trust is up there like 8. When it comes to within the health system, the level of trust will be 6 and for then at the community level it is almost like about 2. For the media I think the level of trust for clinical trials is almost nothing (no trust at all). I think the reason for all these different levels is because of disengagement. It is because Scientists don’t engage much and if people don’t know why, they will ask why, you understand? If I don’t know why you are doing a particular thing, I will ask you why? And because we are not engaging more, we leave a very big loophole in there and for that reason, the media will start chucking (influencing) and say they are hiding, they are hiding, it is not true, they say this vaccine will do this and that, it is not true and if not why are they not coming out clearly? You see it? Then the politicians will also have their own level you know to score political point. For me, I think most Scientists understand the importance of clinical trials because without clinical trials, we cannot be where we are today in terms of health the little thing that the media picks up (say), they turn to believe the media than the Scientists. So even if the community members agree to take part, once the media comes in and say don’t do it they are going to kill you, then they will also follow the media. The reason is that the media is speaking their language and we the Scientists are not speaking their language (KII-48yr old trial coordinator-02).

Reference 2 - 4.64% Coverage

Media influence and

The issue is because they don’t understand the importance of conducting clinical trials,

the little thing that the media picks up (say), they turn to believe the media than the Scientists. So even if the community members agree to take part, once the media comes in and say don’t do it they are going to kill you, then they will also follow the media. The reason is that the media is speaking their language and we the Scientists are not speaking their language.

So I think this work will inform the Scientist that we should not just sit in our conference rooms and talk but then we should go to the grassroots. I remember in one of my trips, in a particular community they wanted to conduct clinical trial and the study team members were knocking on doors, going from house to house and asking people whether they are aware that a trial is going to happen in this hospital and in this community. They were doing that so that those who would say they were not aware of the trial, they would then educate them about the trial and what they were going to do at the hospital. Even in the developed countries, highly educated people, they are still going from door to door and why can’t we do the same here in Ghana? I think it goes back to the grid, the fact that we (refers to Scientists) don’t want to use the money for the people and it is about time we stop that and do the right thing.

<Internals\\KIIs\\A-Clinical trials researchers\\C-KII-58yr old clinical trial investigator and Director-03> - § 2 references coded [6.82% Coverage]

Reference 1 - 3.60% Coverage

With the ebola trial, we had the issues playing around mistrust and this mistrust I think has not been raised by the communities themselves. In my opinion, they themselves were not suspicious but rather information and certain feedbacks that have been given to them by other people who are now created doubts in their minds (KII-58yr old clinical trial investigator and Director-03).

So with the ebola trial and the issue of telephone and so on, I don’t think it was the community that was making noise that oh what is happening here, it was rather when the president got into the seen and said they are taking advantage of poor farmers and enticing them with mobile phones and money and therefore we have to be careful and then the politicians unfortunately who didn’t understand the whole thing (the ebola trial) also jumped into it and say look something is simply wrong and then decided reading politics into it and wondering why they should even do it in this place and not that place and not that it was good and it was bad.

Reference 2 - 3.22% Coverage

The malaria vaccine

With the malaria vaccines, the ground work has been done, the investigators this time around have spoken to Ghana Academy of Arts and Sciences members and they have got their buying in, they have also spoken to the politicians and they have got their buying in and so this thing was ready to go and only for now an independent group somewhere I think outside the country sending whatsapp messages frightening everybody about the vaccine. However, I think this was quickly handled by the researchers because they had already got their communication plan and their crises management plan and so when the issue came up, they just rolled it out and then the issue died off and the trust was reinstalled but then it did bring initial mistrust and concern. People were really scared whether they should send their children or not but that phase is now passed and there is no problem.

<Internals\\KIIs\\A-Clinical trials researchers\\D-KII-50yr old clinical trial investigator-04> - § 1 reference coded [1.79% Coverage]

Reference 1 - 1.79% Coverage

Though as Ghanaian, we have that kind of attitude of pulling people down but apart from that I think we as researchers have not really taken it (refers to clinical trials) as an industry and that is why we are cutting people down, we are killing people and things like that.

<Internals\\KIIs\\A-Clinical trials researchers\\E-KII-48 yr old clinical trial Monitor-05> - § 1 reference coded [7.29% Coverage]

Reference 1 - 7.29% Coverage

Q: Then what was the problem or the reason why the Ebola vaccine trial did not come on and even the current malaria vaccine there are refusal to take the vaccine.

R: The Ebola vaccine was politically motivated, you understand? For the community, the community had accepted until those individuals who wanted to capitalize it for their political ambitions caused that problem. It was an individual who went on air and then the MPs took it up and it became a problem otherwise the community where the trial was to be conducted did not have any problem (KII-48 yr old clinical trial Monitor-05).

The trial was even going on in other countries but for Ghana, it was political and not community issue. The current malaria vaccine the problem also came from outside because some people got videos from a certain lady from UK who circulated it on whatsapp you know painting a bad picture about clinical trials and so it is because of some of these things otherwise for instance those who are refusing, you need to check why you understand? If they are refusing because of these campaigns then it is a different issue because even on USA, there is an NGO that is campaigning against the conduct of clinical trials and so some of these things have started trickling into our communities and so this is what is causing the problem (KII-48 yr old clinical trial Monitor-05).

If not that with our original communities, we generally don’t have challenges when it comes to the conduct of clinical trials.

<Internals\\KIIs\\A-Clinical trials researchers\\G-KII-55yr old clinical trial investigator-07> - § 1 reference coded [6.10% Coverage]

Reference 1 - 6.10% Coverage

Q: Ghana Academy of Arts and Science follows were also against the ebola vaccine trial. What was their main issue?

R: Well, their issues were in line with the issues other people had and so they thought that this was a vaccines that was being tested to prevent the disease and therefore they thought that we were going to bring in the disease with the vaccine to test and see whether the vaccine works or not but who will do that? So the lessons we have learned is that even with the highest level of education people are still very well informed and I think there should be much efforts to inform them about the conduct of clinical trials. So a political disease like ebola and for Ghana academy and sciences to say the kind of things that happened meant that there was lack of communication. The Ghana academy people, who are they? They are made up of professions and doctors from different backgrounds but their knowledge of this particular issue and their information was lacking and also the current methods of vaccine development.

<Internals\\KIIs\\B-Ethics members\\G-KII-54yr old male ethics Ccommitteemember-Nav-01> - § 1 reference coded [7.00% Coverage]

Reference 1 - 7.00% Coverage

Q: Thank you very much Prof. Now, what do you have to say regarding the ebola trial that was suspended in Ghana? What went wrong with that ebola vaccine trial and for it to be suspended?

R: *Yeah*, *eeem*, I think the media kind of put fear on everybody. The way they started the whole thing, they don’t know what is involve in trial studies. They were not properly educated and so when they got the information and the way they communicated the information to Ghanaians scares a lot of people and that made it difficult for people to trust that trial (KII-54yr old male ethics Ccommitteemember-Nav-01).

When something is coming and everybody is talking negative about it, you matter how positive the thing is, people would be highly reluctant to be part of it. So, I think it the *eeeh*, *eeeh*, the media communication was not properly done and therefore, that push people away from even allowing the Scientists to interact with them and explain to them as to why it was important for us to be part of those (refers to ebola) trials. So I think the initial communication was bad and that pushed people away.

<Internals\\KIIs\\B-Ethics members\\I-KII-42yr old female ethics committee member-GHS-03> - § 2 references coded [11.91% Coverage]

Reference 1 - 9.34% Coverage

R: It is all about the education, you know if you are not able to explain your information sheet well to the people (not able to administer informed consent well), if you are not able to do your community entry very well, if you are not able to explain the issues in the language that the person understand to enable them take informed decision then that is where the problem is. So even with the normal protocol, if you are not able to explain the issues for the person to know whether he/she should take part or not then that is where the problem. So the information sheet is very important and it depend on how simple it is and how people are trained to explain the study procedures to the participants to really understand and that is what is going to build the trust. So for instance if you give the information sheet to the person to take home and discuss with another person to probably build on his/her interest then depending on the person’s view on that thing (refers to the study) and what the person has heard before, if there is so many misinformation about it, that is where they might not be trust or something has happened in the community before and they did not understand it well and that is still in their minds and when you go there with a new study, based on previous experience then that is where the trust might not be there.

For instance the Ebola thing (ebola vaccine trial) there were so many hullabaloo (so many noise about it) and people who were even educated did not understand what the study was all about, people did not understand the whole process and when it happens like that and the information gets out there to the community, everyone will interpret it the way he/she wants and that brings about the mistrust (42yr old female ethics committee member-GHS-03)

Reference 2 - 2.57% Coverage

RT: How would you know, they are also individuals before the came together and form the academy, so how did it start? Someone was misinformed first and what I am saying is that it was all about misinformation and if the right information had gotten down and people really understood, it wouldn’t have gotten to that level because they are well educated people. So it had to do with the education because people did not really understand what was going on with regards to that study.

<Internals\\KIIs\\B-Ethics members\\J-KII-59yr old male ethics committee member-GHS-04> - § 1 reference coded [4.22% Coverage]

Reference 1 - 4.22% Coverage

Q: What would you say was the reason why the ebola trial did not take place in Ghana? What were the main concerns?

R: I think for the ebola trial, the first mistake was the media. I think the media came out very early and we did not have strategies to educate them first, to have just called them and explain to them. So I blamed our media because our media is so polarized such that when they get anything everybody wants to sell it and so they come out with non-facts. In this country, they are one of the service industries that have premium place on because of the politicians because they buy them and all of that and so when they say something they (media) carry it out, yeah (KII-59yr old male ethics committee member-GHS-04).

The issue is that we cannot close our boarders and sit down unconcern and we are just lucky that we have not had ebola virus here in Ghana. We should not think that is all because there might be another disease that may cross our boarders and we need these clinical trials to get a good drug to face this thing (refers to disease). Now we know that hand washing is one of the very important things and they might have done this somewhere and that is why they have deplored this strategy.

<Internals\\KIIs\\C-FDA members\\L-KII-35yr old female-FDA-01> - § 2 references coded [9.21% Coverage]

Reference 1 - 5.15% Coverage

R: In Ghana I think the trust is not really with the people in the rural areas, the problem comes with the so called educated people because they sometimes negatively blow things out of proportion and influence people negatively. For instance the ebola vaccine trial, it was our so called MPs and members of Ghana Academy Art and Sciences who made negative noise about the trial. For the people in the rural areas, they have that trust because they usually work with their Physician, their Doctors and they trust that their Doctors will not take undue advantage over them and more so if you have the chance to explain thing to them they understand and so the trust is not with the community members, it is with the so called educated, oh I will say semi-educated because they don’t know everything. They are the once who will usually go to the radio stations and talk about such issue and you would not find rural people doing that. For some time now Clincial trials have been conducting in Ghana and we have never had an issue like the ebola one (trial).

Reference 2 - 4.06% Coverage

Q: So what exactly was the problem with the ebola vaccine trial that was suspended in Ghana? Those who was against the study what was their main problem?

R: I just don’t understand their problem, you see because of freedom of speech and right to information and people are making noise about right to information, right to information, you see sometimes, if the information does not go out in the right way and it goes out in a particular way (wrong way) and it is not explained correctly, it becomes something else and people will blow it out of proportion. So I don’t know whether it is the information that did not go out well or what because as at now I can’t really say exactly what happened. I have the feeling that the information did not go out well and as at now people do not understand what clinical trial is all about.

<Internals\\KIIs\\C-FDA members\\M-KII-38yr old male-FDA-02> - § 3 references coded [14.73% Coverage]

Reference 1 - 3.75% Coverage

Q: You can look at trust ion terms of the stakeholders at the national level and also trust of community members to take part in these trials.

R: Well I think people have the trust in clinical trials that have been conducted. On the issue of the educated people, they read and also listen to what goes on and people who are against clinical trials have their own mission of actually discouraging people from taking part trials or vaccine studies and all that and you will always have those distractors but I think that most people believe in the state institutions such as the FDA that regulate clinical trial because they know they will always do a good job.

Reference 2 - 7.21% Coverage

But, you see what the problem is has to do with the communication and it is not the fault of the regulator because before we approve the study you cannot make noise about the trial, you cannot go ahead to even do education on it that there is going to be a trial because what if you do not get approval? And that will really put pressure on the regulator and we don’t normally encourage researchers to go ahead and do education or advertise it before the approval is given. When you do that it puts pressure on the regulator and what if it is not approved? So the ebola information actually got to the general public before the approval and the journalists also took it up and turned the whole thing and for some of the people we taught were educated also made matters worse. So it is more or less the communication, how we communicate some of these things and in the case of the ebola trial, the information got outside there and the journalists took it up before the approval from FDA and they changed everything about the trial, for instance giving people mobile phones to influence them to take part and all that but the phones were to be given to them for follow-ups. So if you don’t get the correct picture, you would not understand the whole things, yes.

Reference 3 - 3.76% Coverage

Q: Is like there is also a problem about the malaria vaccine and I dot know whether you have heard about that?

R: Yes, but the point is that they are always there, those people who are against vaccination are there and they will always speak against any new vaccine that is coming into the country. I remember before the rotavirus vaccine was implemented in 2012, we had people who spoke against it but finally when the rotavirus trial came in it had reduced a lot of deaths and that trial was conducted in Navrongo. So you have those people and the solution is to just manage them and I think we should have a good communication plan, which we should implement.

**Name:** uncertainty of treatment effectiveness

<Internals\\KIIs\\A-Clinical trials researchers\\B-KII-48yr old trial coordinator-02> - § 1 reference coded [2.90% Coverage]

Reference 1 - 2.90% Coverage

Q: Do you also think that doubts and uncertainties about these new drugs and the use of placebo in trial studies also negatively affect trust of community members?

R: Of course, because at times when you don’t make things clear to people, it brings doubts and when doubts comes in then people will start questioning. So the issue is that most at times what we tell them is that we are going to give you drug and this drug will do this or that but with the placebo, we don’t tell them about it for them to understand that maybe you are likely not to get the real drug. I don’t know whether it is usually the design of the study and I have not seen any design that says that don’t tell them because if you are telling them about the drug itself, you should be able to tell them something about the placebo because they are supposed to know everything for them to take a decision.
